# Supplementary material for: Naive Human Embryonic Stem Cells Can Give Rise to Cells with a Trophoblast-like Transcriptome and Methylome
Source: Stem Cell Reports. 2020 Jul 2;15(1):198–213. doi: 10.1016/j.stemcr.2020.06.003 (PMC7363941; doi:10.1016/j.stemcr.2020.06.003)
Supplement: Document S1. Supplemental Experimental Procedures and Figures S1–S7 [file mmc1.pdf]

Stem Cell Reports, Volume *15*

Supplemental Information

Naive Human Embryonic Stem Cells Can Give Rise to Cells with a Trophoblast-like Transcriptome and Methylome

Jessica K. Cinkornpumin, Sin Young Kwon, Yixin Guo, Ishtiaque Hossain, Jacinthe Sirois, Colleen S. Russett, Hsin-Wei Tseng, Hiroaki Okae, Takahiro Arima, Thomas F. Duchaine, Wanlu Liu, and William A. Pastor

Figure S1

A.

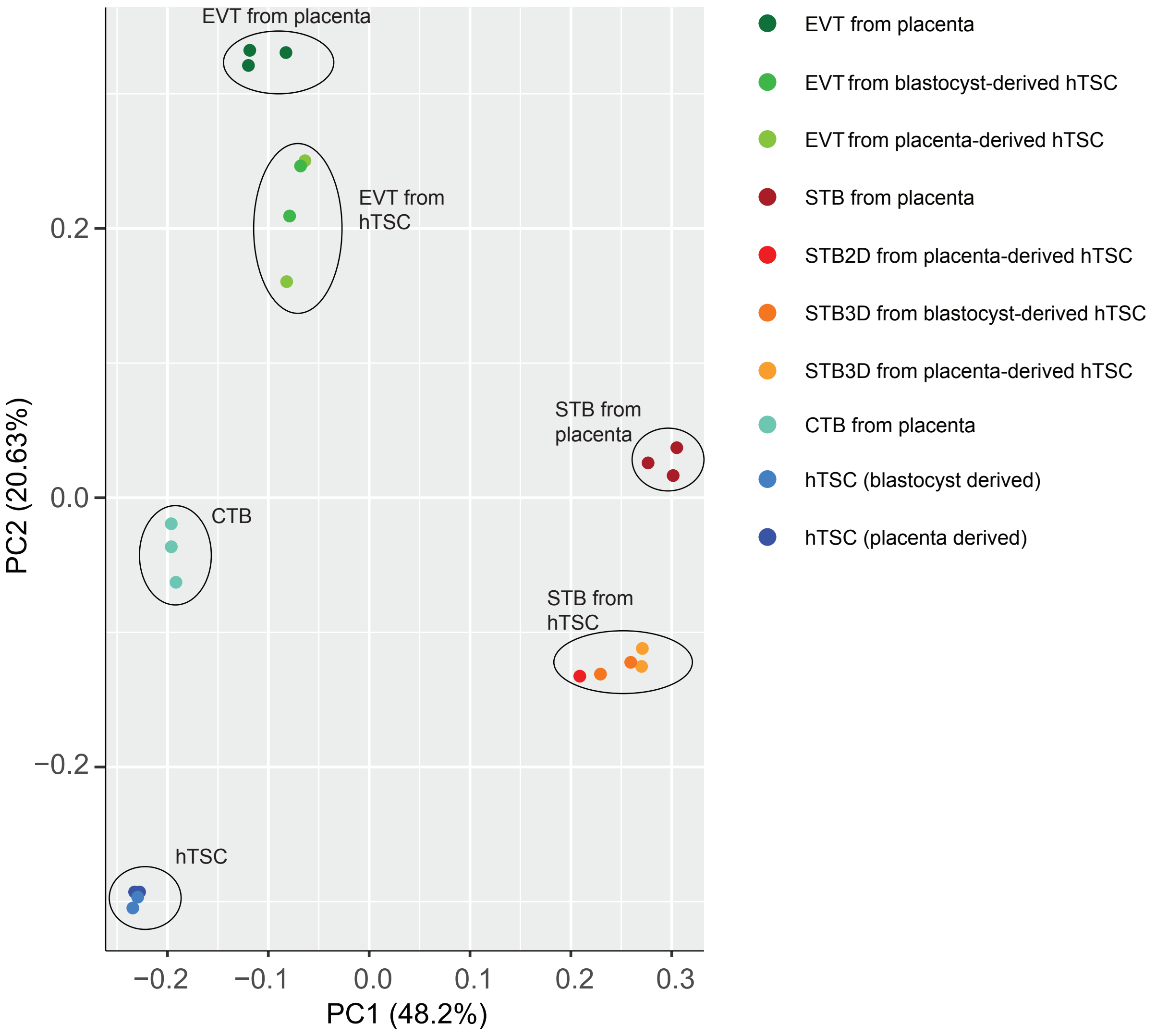
EVT from placenta
EVT from hTSC
STB from placenta
CTB
STB from hTSC
hTSC
PC2 (20.63%)
PC1 (48.2%)
0.2
0.0
−0.2
−0.2
−0.1
0.0
0.1
0.2
0.3
EVT from placenta
EVT from blastocyst-derived hTSC
EVT from placenta-derived hTSC
STB from placenta
STB2D from placenta-derived hTSC
STB3D from blastocyst-derived hTSC
STB3D from placenta-derived hTSC
CTB from placenta
hTSC (blastocyst derived)
hTSC (placenta derived)

B.

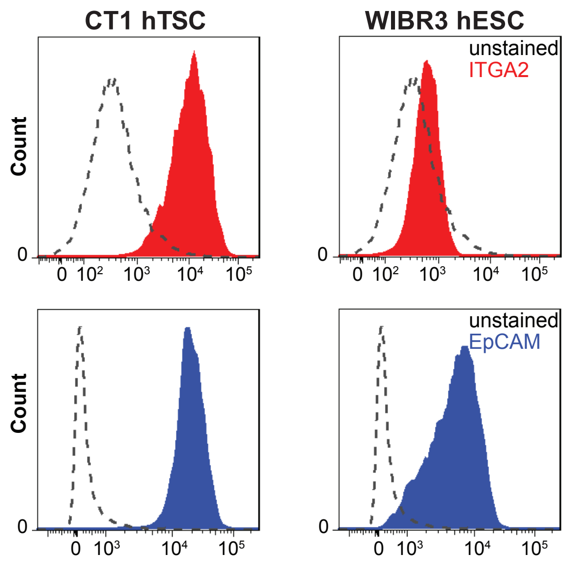
CT1 hTSC
WIBR3 hESC
unstained
ITGA2
unstained
EpCAM
Count
Count

Figure S1. Principle component analysis of gene expression of different placental cell types. Related to Figure 1. **A.** Principle component analysis was performed using RNA-seq data from (Okae et al., 2018). Cells analyzed include cell types isolated directly from placenta (CTB = cytotrophoblast, STB= syncytiotrophoblast, EVT=extravillous trophoblast) and lines cultured and differentiated *in vitro*. These include blastocyst-derived hTSC lines (BT1 and BT2), placental-derived hTSC lines (CT1 and CT2), as well as EVT and STB derived from these lines. "2D" and "3D" STB refer to two differentiation protocols. **B.** Flow cytometry for ITGA2 and EpCAM in hTSCs and hESCs. Staining profile is indicated relative to unstained control.

Figure S2

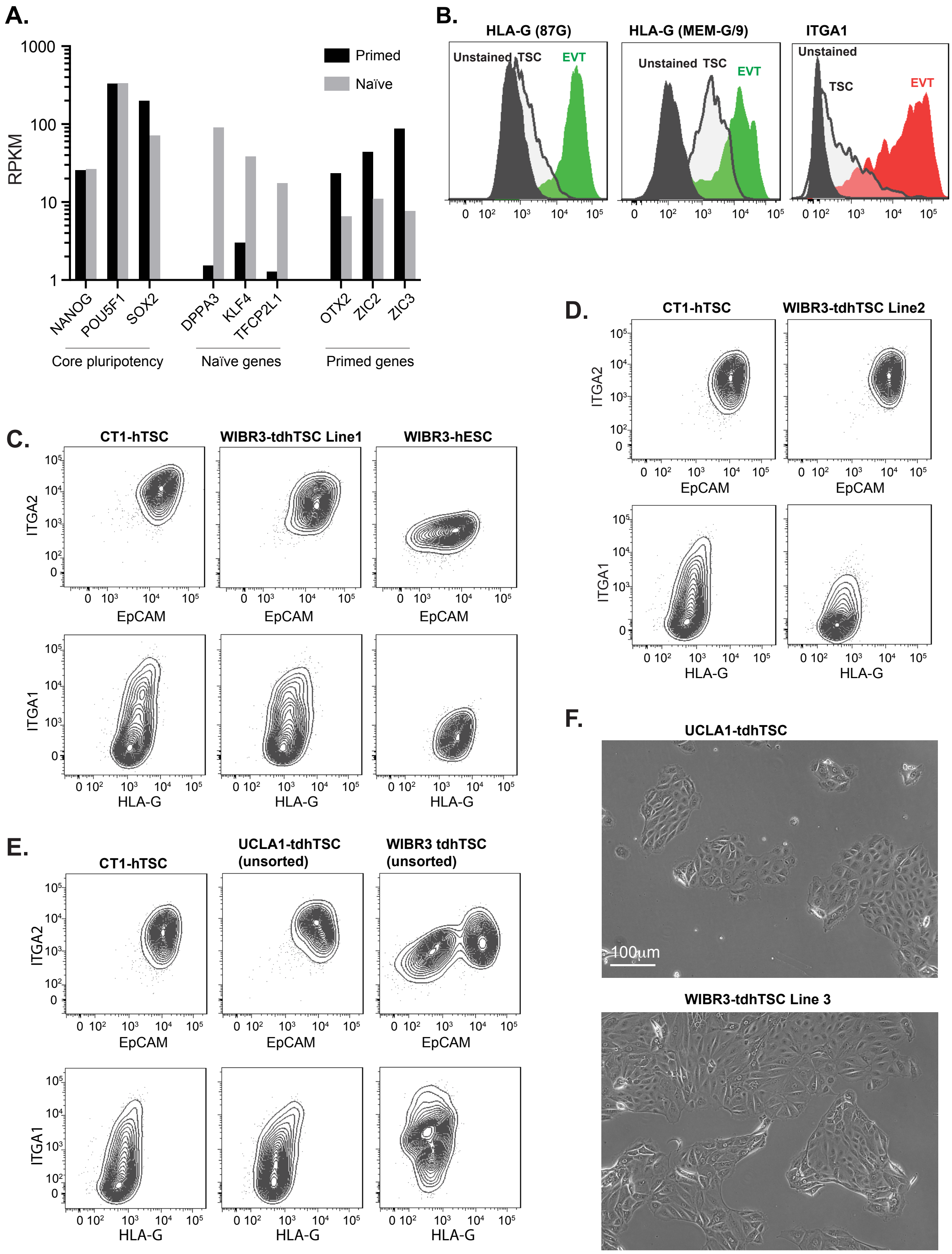

A.
B.
C.
D.
E.
F.
RPKM
Primed
Naïve
NANOG
POU5F1
SOX2
DPPA3
KLF4
TFCP2L1
OTX2
ZIC2
ZIC3
Core pluripotency
Naïve genes
Primed genes
HLA-G (87G)
HLA-G (MEM-G/9)
ITGA1
Unstained
TSC
EVT
CT1-hTSC
WIBR3-tdhTSC Line1
WIBR3-hESC
WIBR3-tdhTSC Line2
UCLA1-tdhTSC (unsorted)
WIBR3 tdhTSC (unsorted)
ITGA2
ITGA1
EpCAM
HLA-G
UCLA1-tdhTSC
WIBR3-tdhTSC Line 3
100μm

Figure S2. Transdifferentiation of hESCs to putative hTSCs and purification via FACS sorting. Related to Figure 2. **A.** Expression of primed and naïve hESC markers in primed and Day 10 naïve cells (which were used as the starting point for generation of WIBR3 tdhTSC Line 1) as measured by RNA-sequencing of one replicate. **B.** Flow cytometry validation of HLA-G and ITGA1 antibodies used in other figures. Note elevated HLA-G and ITGA1 signal in EVT. Representative of n= 5 – 15 independent experiments per antibody **C.** Flow cytometry of WIBR3 tdhTSC Line 1, 16 days after sorting, with CT1 and WIBR3 hESCs as comparisons. Representative of n= 2 (hESCs), and n=3 (WIBR3 tdhTSC L1) independent flow cytometry experiments conducted with these lines. **D.** Flow cytometry profile of WIBR3 tdhTSC Line 2. Representative of n= 3 independent experiments. **E.** UCLA1 and WIBR3 hESCs were reverted and transferred to hTSC media, with flow cytometry conducted 7 days later. Note formation of $ITGA2^{hi}$ $EpCAM^{hi}$ $ITGA1^{lo}$ cells in both lines. Same CT1 control was used in D. and E. **F.** Photographs of sorted $ITGA2^{hi}$ $EpCAM^{hi}$ $ITGA1^{lo}$ UCLA1 tdhTSC Line 1 and WIBR3 tdhTSC Line 3.

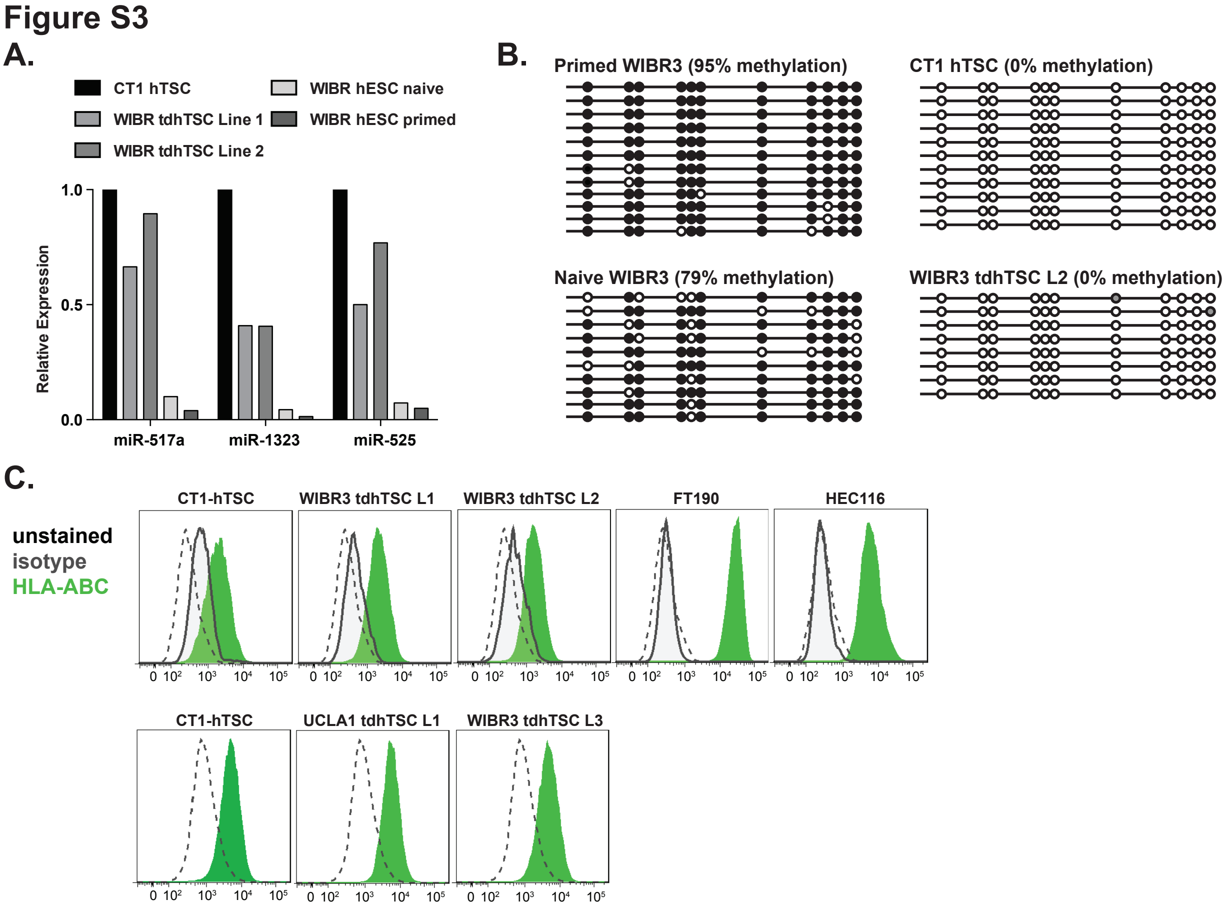

Figure S3. Transdifferentiated hTSCs show hallmarks of placental identity. Related to Figure 3. **A.** RT-PCR of three miRNA that are generated from the placental C19MC transcript. n=1 biological replicate. **B.** Bisulfite PCR of the ELF5 locus for the Primed, Naïve and WIBR3 tdhTSC L2, as well as control CT1 line. Each CG site is indicated with a circle, with an empty circle indicating an unmethylated CG, a black circle indicating methylation, and a gray circle indicating non-informative sequencing. Note modest demethylation in naïve culture and dramatic demethylation upon transdifferentiation. **C.** Flow cytometry for a pan-HLA antibody in CT1, WIBR3 tdhTSC Lines 1 and 2, FT190 and Hec116. Unstained, isotype control, and pan-HLA staining profiles are indicated. Representative of n=2 independent experiments.

Figure S4

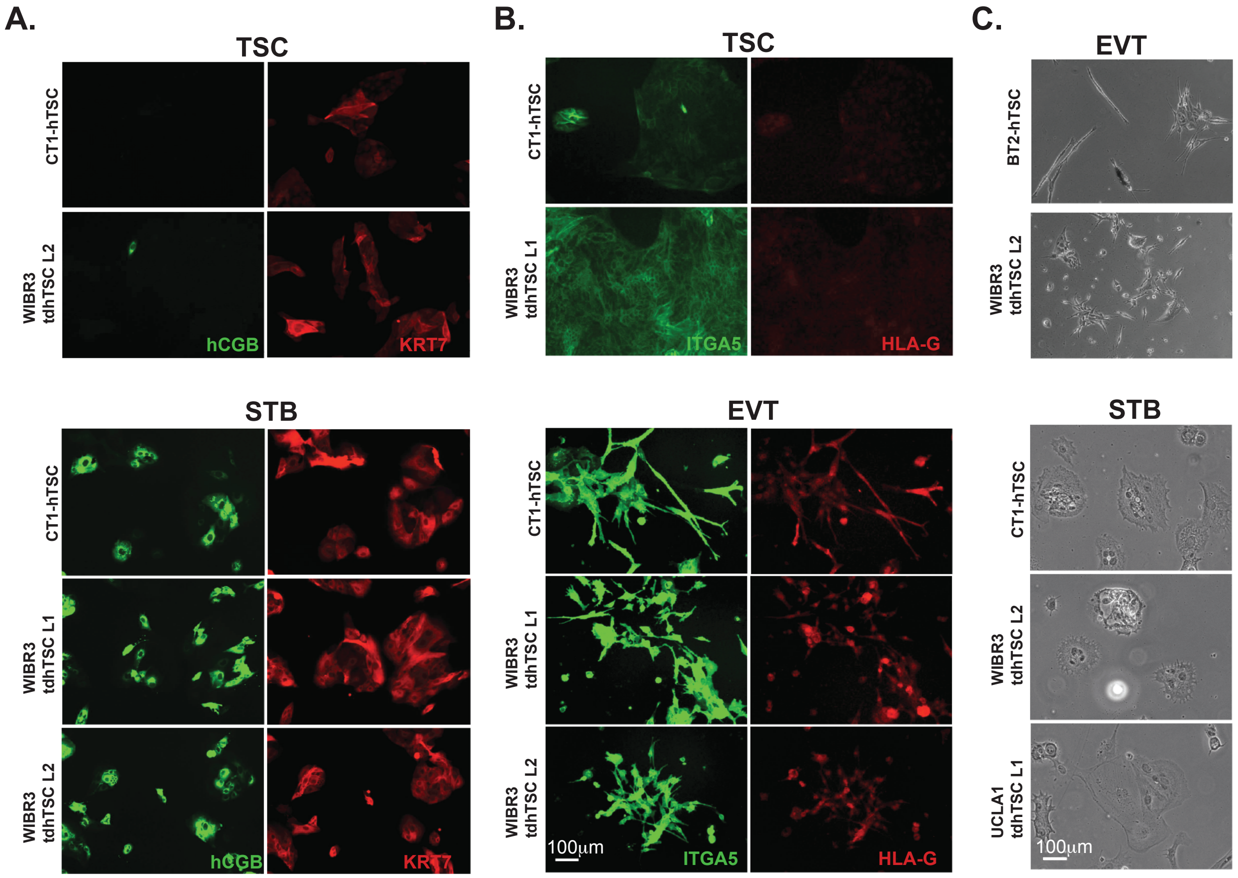

Figure S4. Differentiation capacity of tdhTSCs. Related to Figure 4. **A,B.** Immunofluorescent staining of cells indicated with STB marker (hCGB), EVT markers (ITGA5, HLA-G) or a pan-placental marker (KRT7). Note gain of hCGB upon STB differentiation **(A)** and gain of spindly mesenchymal morphology and increased staining for ITGA5 and HLA-G upon EVT differentiation **(B). C.** Light microscopy photos of EVTs and STBs indicated.

Figure S5

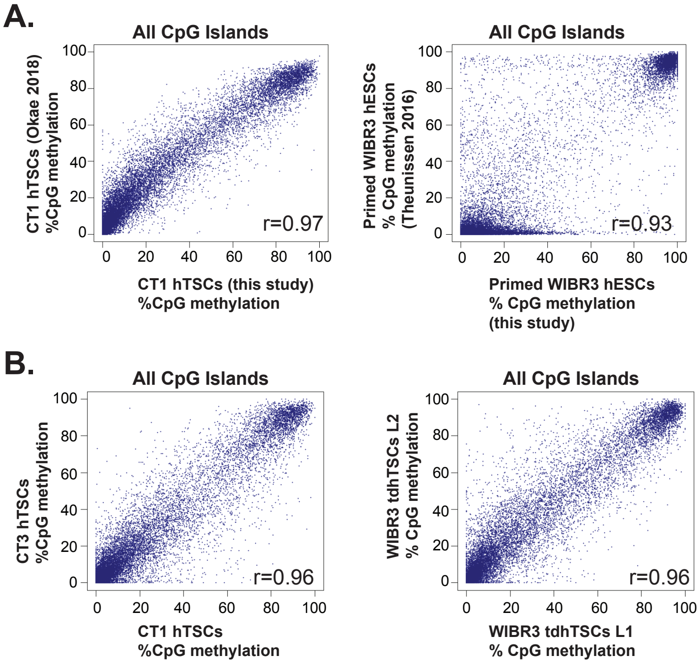

Figure S5. Global methylation patterns of tdhTSC. Related to Figure 5. **A.** Scatterplot showing CpG island methylation for each CpG island in our sample (X-axis) and published data (Y-axis). Note high correlation between new and published data. **B.** Scatterplot showing CpG island methylation for similar samples (CT1 vs. CT3, WIBR3 tdhTSC L1 vs. L2).

Figure S6

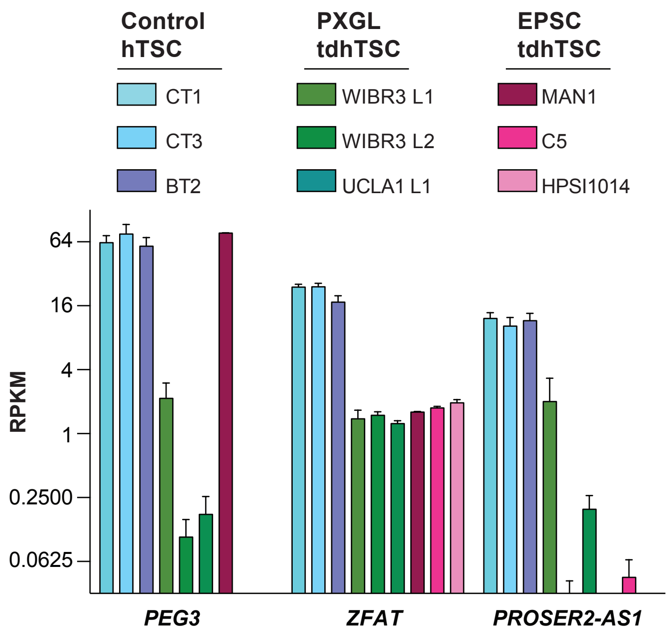

Figure S6. Imprinting abnormalities in tdhTSC. Related to Figure 6. **A.** Expression of the three imprinted genes indicated in control and tdhTSCs. Data for EPS tdhTSCs are from published sources(Gao et al., 2019). n=2 (all tdhTSC lines), n=4 (CT3, BT2) or n=7 (CT1) biological replicates.

Figure S7

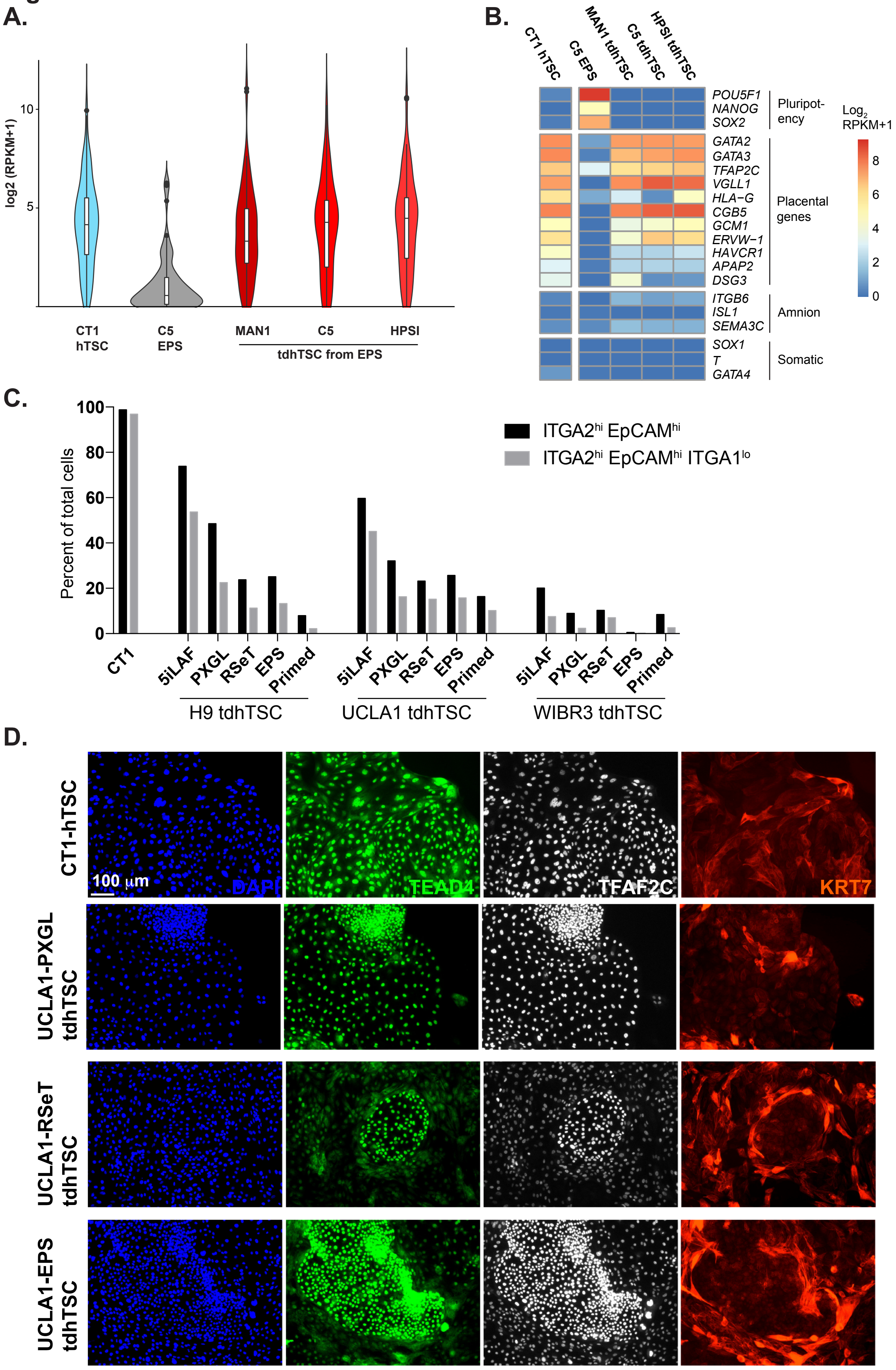
A.
log2 (RPKM+1)
10
5
CT1
hTSC
C5
EPS
MAN1
C5
HPSI
tdhTSC from EPS
B.
CT1 hTSC
C5 EPS
MAN1 tdhTSC
C5 tdhTSC
HPSI tdhTSC
POU5F1
NANOG
SOX2
Pluripot-
ency
GATA2
GATA3
TFAP2C
VGLL1
HLA−G
CGB5
GCM1
ERVW−1
HAVCR1
APAP2
DSG3
Placental
genes
ITGB6
ISL1
SEMA3C
Amnion
SOX1
T
GATA4
Somatic
Log2
RPKM+1
8
6
4
2
0
C.
Percent of total cells
100
80
60
40
20
0
ITGA2hi EpCAMhi
ITGA2hi EpCAMhi ITGA1lo
CT1
5iLAF
PXGL
RSeT
EPS
Primed
H9 tdhTSC
UCLA1 tdhTSC
WIBR3 tdhTSC
D.
CT1-hTSC
UCLA1-PXGL
tdhTSC
UCLA1-RSeT
tdhTSC
UCLA1-EPS
tdhTSC
100 μm
DAPI
TEAD4
TFAF2C
KRT7

Figure S7. Comparative transdifferentiation capacity from different media conditions. Related to Figure 7. **A.** Expression of 89 placental genes (same as in Figure 3D) in tdhTSCs derived from EPS cells. Expression of each gene, using an average of all replicates for a given cell type, is indicated as a single point on the violin plot. n=2 (EPS and tdhTSC) or n=7 (CT1) biological replicates. Data for EPS cells and EPS tdhTSCs are from published sources (Gao et al., 2019). **B.** Expression of markers indicated from RNA-seq data. Data for EPS cells and EPS tdhTSCs are from published sources (Gao et al., 2019). Data from n=2 (EPS and tdhTSC) or n=7 (CT1) biological replicates are averaged. **C.** Percentage of $ITGA2^{hi}$ $EpCAM^{hi}$ and $ITGA2^{hi}$ $EpCAM^{hi}$ $ITGA1^{lo}$ cells after hESCs of the line and starting conditions indicated are cultured in hTSC media for 15 days. n=1 experiment. **D.** Immunofluorescent images of control CT1 hTSCs and tdhTSCs derived from hESCs cultured in naïve (PXGL) and primed-like (EPS, RSeT) conditions. Note uniform staining for the three indicated markers in CT1 and PXGL-derived hTSCs, but a mixture of $TEAD4^{hi}$ and $TEAD4^{lo}$ cells in EPS or RSeT derived tdhTSCs. n=1 experiment.

Supplemental Table Captions

Table S1. STR analysis of WIBR3 hESCs and TSC. Related to Figure 2.

Table S2. Descriptions of transdifferentiations. Related to Figures 2 and 7. Description of circumstances of generation of each cell line, efficiency of production of ITGA2^{hi} EpCAM^{hi} and ITGA2^{hi} EpCAM^{hi} ITGA1^{lo} cells, and figures in which each cell line was used.

Table S3. Sample description and mapping statistics. Related to Figures 3, 5, 6.

Table S4. RPKM of all samples. Related to Figures 3, 5, 6.

Table S5. Expression of trophoblast-specific genes in RNA-seq samples. Related to Figure 3. Expression data used to generate Figure 3D.

Table S6. Methylation data. Related to Figures 5,6. CpG islands with CT1-hTSC-specific methylation, promoters with hESC-specific methylation, and putative gatekeeper genes and their expression levels in different samples are all shown.

Table S7. Genes differentially expressed between control hTSC and tdhTSC. Related to Figure 6. First two panels: Genes that show differential expression between hTSCs (CT1, CT3, BT2) and primed hESCs (WIBR3, UCLA1) are listed, along with fold-change and log_2 read count per million. Positive fold-change value indicates higher expression in hESCs. Third and fourth panels: Genes that show differential expression between control hTSCs (CT1, CT3, BT2) and tdhTSCs (WIBR-tdhTSC Line 1, WIBR3-tdhTSC Line 2, UCLA1 tdhTSC) are listed. Positive fold-change value indicates higher expression in tdhTSCs.

Supplemental Experimental Procedures

Naïve Reversion: Primed hESCs were routinely passaged in TeSR-E8 media (Stemcell Technologies). In the first naïve reversion we conducted (which led to the generation of WIBR3 tdhTSC L1 and L2), primed hESCs were cultured in special primed media (DMEM-F12 supplemented with 15% FBS, 5% KSR, 1X Glutamax, 1X non-essential amino acids, 0.1mM β-mercaptoethanol and 8 ng/ml FGF) on mitomycin C inactivated mouse embryonic fibroblast (MEF) feeder layers for one passage prior naïve reversion. In subsequent reversions, cells were cultured in TeSR-E8 until naïve induction.

In accordance with published protocol (Guo et al., 2017) 200,000 primed hESCs were dissociated into single cells using 30% TrypLE Express and plated onto a MEF feeder layer in special primed media (in the first reversion) or TeSR E8 (in subsequent reversions) supplemented with 10 μM ROCKi at 5% O_2. After 24h, primed hESCs were cultured for 3 days in naïve induction medium (24.5 ml DMEM F-12, 24.5 ml Neurobasal media, 0.25 ml N2, 0.5 ml B27, 1x Glutamax, 150 μM L-ascorbic acid, 0.1mM β-mercaptoethanol, 1μM PD0325901, 10μM ROCKi, 0.75mM vaporic acid and 10ng/ml recombinant hLIF). Following induction, reverting naïve hESCs were cultured in PXGL maintenance medium (24.5 ml DMEM F-12, 24.5 ml Neurobasal media, 0.25 ml N2, 0.5 ml B27, 1xGlutamax, 150 μM L-ascorbic acid, 0.1mM β-mercaptoethanol, 1μM PD0325901, 2μM Gö6983, 10μM ROCKi, 2μM XAV939 and 10ng/ml recombinant hLIF) indefinitely. Reverting naïve cells started showing dome-like morphology with refractive edges 7 days post-induction. We observed that culture with ascorbate (described as optional in previous protocols(Guo et al., 2016)) enhanced subsequent transdifferentiation (data not shown).

Culture of RSet cells was conducted with a commercially available reagent (Stemcell Technologies 05975) and manufacturer protocol. EPS cells were generated and cultured by published protocol(Gao et al., 2019). Cells were cultured in RSeT or EPS media for 11 days before culture in hTSC media.

Flow cytometry: Cells were dissociated with TrypLE Express (Gibco 12604) and quenched with Soybean trypsin inhibitor (Gibco 17075), then passed over a 70µm filter to remove aggregates. Cells were then centrifuged 3 minutes at 200xg and resuspended in 1ml FACS buffer (1xPBS, 1%BSA) and counted.

Cells were stained with appropriate fluorescent antibodies at a concentration of 1µg antibody per one million cells in a volume of 1ml. When fewer cells were used, antibody and volume were scaled down accordingly. Cells were incubated with antibody for 20 minutes in the dark at 4°C, then centrifuged, washed with 1ml of FACS buffer, resuspended in 300µl FACS buffer and analyzed. DAPI was included immediately before flow to distinguish dead cells. Flow cytometry was performed using a BD FACSAria Fusion instrument, FACS with a BD LSRFortessa instrument, and analysis was performed using FlowJo v10.

A table indicating which antibodies were used and in which figures is shown below:

EPCAM conj. BV711	Biolegend	324239	Figure 1C, 2E, 2H, 7A, 7B, S1B, S2C-E
ITGA1 (Integrin alpha 1) conj. APC	Biolegend	328313	Figure 2E, 2H, 4C, S2B-E
ITGA2 (Integrin alpha 2) conj. 594	R&D	FAB1233T	Figure 1C, 2E, 2H, 7A, 7B, S1B, S2C-E
ITGA6 (Integrin alpha 6) conj. APC/Fire 750	Biolegend	313631	Figure 7B
ITGB6 (Integrin beta 6) conj. APC	Miltenyi	130-111-454	Figure 7B,C
HLA-G (Major histocompatibility complex-G) conj. 488	Biolegend	335917	Figure 2E, 2H, S2B-E
HLA-G (Major histocompatibility complex-G) conj. PE	Abcam	24384	Figure 4C, S2B
HLA-ABC (Major histocompatibility complex-ABC) conj. 488	Biolegend	311415	Figure S3C
IgG anti-mouse	Cell signalling	61656	Figure S3C

Immunofluorescence*:* Cells were plated and grown on glass coverslips coated with extracellular matrix medium appropriate for the cell type or differentiation. Cells were fixed in 4% PFA, 0.75xPBS for 15 minutes at room temperature, then washed twice with 1xPBS. They were then permeabilized and blocked by treatment with blocking buffer (0.1% Triton X, 5% Donkey serum, 1xPBS) for 30 minutes, then washed twice with 1xPBS 0.05% Tween-20. Cells were incubated with indicated concentration of primary antibody in blocking buffer for 1-2 hours at room temperature. Cells were then washed twice with 1xPBS 0.05% Tween-20. Secondary antibody incubation was performed by incubation for one hour with Invitrogen ALEXA-Fluor antibodies. Cells were washed once more with 1xPBS 0.05% Tween-20 containing DAPI (Sigma), then mounted with Invitrogen Prolong GOLD to preserve the imaging. Images were taken with Zeiss-Axiovert or Invitrogen-EVOS and fluorescence was adjusted uniformly across experiments.

Antibodies and respective concentrations are indicated below:

ITGA5 (Integrin alpha 5)	dilution: 1:1000	Abcam	150361
hCGB (human Chorionic Gonadotropin)	dilution: 1:1000	Abcam	131170
KRT7 (Cytokeratin 7) conj. 594	dilution: 1:500	Biolegend	601603
HLA-G (Major histocompatibility complex) conj.PE	dilution: 1:500	Abcam	24384
TFAP2C (Transcription factor AP-2)	dilution: 1:1000	Abcam	76007
TEAD4 (Transcription enhancer factor)	dilution: 1:500	Abcam	58310

Real time PCR analysis of gene expression: RNA isolation was performed using the RNAzol total RNA protocol (Sigma). cDNA synthesis was performed using Froggabio SensiFAST cDNA synthesis kit using 500ng total RNA/10μl reaction following manufacturer settings. Each reaction mix is then diluted to 50 μl with Rnase DNase free H_2O. 1μL of each cDNA reaction mix, corresponding to 10ng of starting RNA, was used in each 15 μl reaction of Invitrogen PowerUp SYBR green mix containing 1μM of primer mix. Quantification and analysis were performed on the QuantStudio5 instrument.

Primers:

ITGA2:	F:5'-ATGAAAACCAACATGAGCCTCG-3'	R:5'-GATTCCCACATTGCTGTGCC-3'
GCM1:	F:5'-TGAACACAGCACCTTCCTCC-3'	R:5'-CGCCTTCCTGGAAAGACCAA-3'
HLA-G:	F:5'-GCCAAGGATGGTGGTCATGG-3'	R:5'-GCGGCGCTGAAATACCTCAT-3'
CGB7:	F:5'-CGCACCAAGGATGGAGATGT-3'	R:5'-GAAGCATCTCCCTGGATGCC-3'
TFAP2C	F-5'- TGCACGATCAGACAGTCATTC	R-5'-GTAGAGCTGAGGAGCGACAATC-3'
GATA3	F-5'- TGCAGGAGCAGTATCATGAAGCCT -3'	R-5'-GCATCAAACAACTGTGGCCAGTGA-3'
XAGE3	F-5'-CCGAGGAGAAGTGTACCACC-3'	R-5'-TGCAGGATCCCGACTTTCAG-3'
HAVRC1	F-5'-GGCGTATATTGTTGCCGTGT-3'	R-5'-GACGGTTGGAACAGTTGTGA-3'
ELF5	F-5'- GACGCTGAAGAAAGCAAGGC-3'	R-5'- CCCATTCCAGAATGCCACAG-3'
ITGB6	F-5'-GAAGAAATTGCCAACCCTTG-3'	R-5'TGTCAATGGCAAAATGTGCT-3'

The TFAP2C, GATA3 and ELF5 primers were taken from published sources(Lee et al., 2016).

ELF5 Methylation analysis: 500 ng of genomic DNA was bisulfite-converted using EZ DNA Methylation-Lightning Kit (Zymo) as per instructions. Converted DNA was eluted in 10 μL of M-elution buffer.

The ELF5 promoter was amplified using primers described previously(Lee et al., 2016). We used a nested PCR strategy in which the locus was first amplified with primer set #1 (forward:5'-GGAAATGATGGATATTGAATTTGA-3', reverse:5'-CAATAAAAATAAAAACACCTATAACC-3'). Each 20 μL PCR reaction was carried out using 10 μl 2X Zymotaq premix, 0.5 μl forward primer, 0.5 μl reverse primer, 8 μL H20, and 1 μL converted DNA, with PCR cycling conditions: 95°C 10:00, 35x (95°C 30s, 45°C 30s, 72°C 30s), 72°C 7:00). 1μl of the reaction product was then amplified using identical conditions and cycles with a second set of primers (forward:5'-GAGGTTTTAATATTGGGTTTATAATG-3', reverse:5'-ATAAATAACACCTACAAACAAATCC-3').

PCR products were purified with a MinElute PCR purification kit (Qiagen), using manufacturer protocols with the addition of a second elution step in which the eluant is passed over the column a second time. PCR product was cloned into pGEM-T Easy Vector (Promega) using a 1:3 plasmid:insert molar ratio, transformed into DH5α competent bacteria, cultured and miniprepped using the Presto Mini Plasmid Kit (FroggaBio).

C19 microRNA cluster expression analysis: Total RNA was extracted using RNAzol RT (Bioshop) followed by isopropanol precipitation. DNase treatment was then performed on all the total RNA samples with TURBO DNase (Thermo Fisher) following manufacturer's instructions. To quantify mature miRNAs, the miScript kit (Qiagen) was used for reverse transcription using 500ng of total RNA input and diluted 1 in 4 for subsequent RT-PCR reactions. QuantiTect SYBR Green PCR kit (Qiagen) was used for miRNA quantification, with customized forward primers for miRNAs, primer assay for the control U6 snRNA (Qiagen MS00033740), and the kit supplied universal primer as the reverse primer. The cycling condition for RT-PCR is: (95°C 15 min, 45x (94°C 15 s, 55°C 30 s, 70°C 30 s), 95°C 15 s). The following primers were used:

miR-1323: TCAAAACTGAGGGGCATTTTCT

miR-525-5p: CTCCAGAGGGATGCACTTTCT
miR-517a-3p: ATCGTGCATCCCTTTAGAGTGT

Supplemental References

Gao, X., Nowak-Imialek, M., Chen, X., Chen, D., Herrmann, D., Ruan, D., Chen, A.C.H., Eckersley-Maslin, M.A., Ahmad, S., Lee, Y.L., *et al.* (2019). Establishment of porcine and human expanded potential stem cells. Nat Cell Biol *21*, 687-699.

Guo, G., von Meyenn, F., Rostovskaya, M., Clarke, J., Dietmann, S., Baker, D., Sahakyan, A., Myers, S., Bertone, P., Reik, W., *et al.* (2017). Epigenetic resetting of human pluripotency. Development *144*, 2748-2763.

Guo, G., von Meyenn, F., Santos, F., Chen, Y., Reik, W., Bertone, P., Smith, A., and Nichols, J. (2016). Naive Pluripotent Stem Cells Derived Directly from Isolated Cells of the Human Inner Cell Mass. Stem Cell Reports *6*, 437-446.

Lee, C.Q., Gardner, L., Turco, M., Zhao, N., Murray, M.J., Coleman, N., Rossant, J., Hemberger, M., and Moffett, A. (2016). What Is Trophoblast? A Combination of Criteria Define Human First-Trimester Trophoblast. Stem Cell Reports *6*, 257-272.

Okae, H., Toh, H., Sato, T., Hiura, H., Takahashi, S., Shirane, K., Kabayama, Y., Suyama, M., Sasaki, H., and Arima, T. (2018). Derivation of Human Trophoblast Stem Cells. Cell Stem Cell *22*, 50-63 e56.
