# Supplementary material for: Naive Human Embryonic Stem Cells Can Give Rise to Cells with a Trophoblast-like Transcriptome and Methylome
Source: Stem Cell Reports. 2020 Jul 2;15(1):198–213. doi: 10.1016/j.stemcr.2020.06.003 (PMC7363941; doi:10.1016/j.stemcr.2020.06.003)
Supplement: Document S2. Article plus Supplemental Information [file mmc9.pdf]

Stem Cell Reports
Article
OPEN ACCESS

Naive Human Embryonic Stem Cells Can Give Rise to Cells with a Trophoblast-like Transcriptome and Methylome

Jessica K. Cinkornpumin,[1] Sin Young Kwon,[1] Yixin Guo,[4] Ishtiaque Hossain,[1] Jacinthe Sirois,[1,2] Colleen S. Russett,[1] Hsin-Wei Tseng,[1] Hiroaki Okae,[5] Takahiro Arima,[5] Thomas F. Duchaine,[1,2] Wanlu Liu,[3,4] and William A. Pastor[1,2,*]
[1]Department of Biochemistry, McGill University, Montreal, QC H3G 1Y6, Canada
[2]The Rosalind & Morris Goodman Cancer Research Centre, McGill University, Montreal, QC H3A 1A3, Canada
[3]Department of Orthopedic of the Second Affiliated Hospital of Zhejiang University School of Medicine, Zhejiang University, Hangzhou 310029, China
[4]Zhejiang University-University of Edinburgh Institute (ZJU-UoE Institute), Zhejiang University School of Medicine, International Campus, Zhejiang University, 718 East Haizhou Road, Haining 314400, China
[5]Department of Informative Genetics, Environment and Genome Research Centre, Tohoku University Graduate School of Medicine, Sendai 980-8575, Japan
*Correspondence: william.pastor@mcgill.ca
https://doi.org/10.1016/j.stemcr.2020.06.003

SUMMARY

Human embryonic stem cells (hESCs) readily differentiate to somatic or germ lineages but have impaired ability to form extra-embryonic lineages such as placenta or yolk sac. Here, we demonstrate that naive hESCs can be converted into cells that exhibit the cellular and molecular phenotypes of human trophoblast stem cells (hTSCs) derived from human placenta or blastocyst. The resulting "transdifferentiated" hTSCs show reactivation of core placental genes, acquisition of a placenta-like methylome, and the ability to differentiate to extravillous trophoblasts and syncytiotrophoblasts. Modest differences are observed between transdifferentiated and placental hTSCs, most notably in the expression of certain imprinted loci. These results suggest that naive hESCs can differentiate to extra-embryonic lineage and demonstrate a new way of modeling human trophoblast specification and placental methylome establishment.

INTRODUCTION

In most mammals, the first cellular specification event is believed to be acquisition of placental or non-placental identity (Pfeffer, 2018). Distinct polarized outer and apolar inner cell populations form during the morula phase of development. In the subsequent blastocyst stage, the outer cells give rise to the trophoblast lineage and later form most cells in the placenta. The inner cells give rise to the inner cell mass, which specifies both the hypoblast and pluripotent epiblast. The epiblast generates all embryonic tissues (ectoderm, mesoderm, and endoderm) of the organism. The observations above have been demonstrated by lineage tracing, blastomere transplantation, and chimera experiments in mice (Chazaud and Yamanaka, 2016). On the basis of observation, immunofluorescent staining, and RNA sequencing (RNA-seq), it is likely that human development follows a similar pattern (Niakan et al., 2012; Stirparo et al., 2018).

After implantation of the blastocyst into the uterine wall, the trophoblast lineage develops rapidly. Structures called villi sprout and expand. Cytotrophoblasts (CTBs), a population of epithelial cells within the villi, fuse to form syncytiotrophoblasts (STBs), large multinucleated cells that line the surface of the villi, secrete pregnancy hormones, and mediate gas and nutrient exchange with maternal blood. At the tips of villi that contact maternal tissue, CTBs undergo epithelial to mesenchymal transition and differentiate into extravillous trophoblasts (EVTs). EVTs invade maternal tissue, expand and anchor the villi, and remodel maternal arterioles (Maltepe and Fisher, 2015).

Meanwhile, the epiblast undergoes a series of changes, including epithelialization, increased DNA methylation, and expression of a new set of genes and cell surface receptors. These changes prime the epiblast to differentiate rapidly in response to external cues during subsequent gastrulation. As such, the epiblast is said to transition from the "naive" pluripotent state to the "primed" pluripotent state (Nichols and Smith, 2009). Upon gastrulation, the epiblast differentiates, and pluripotency is lost.

Stem cells have been used to study these developmental stages in mice and humans. In mice, embryonic stem cells (ESCs) can be cultured from the blastocyst inner cell mass, exhibit naive pluripotency, and can be differentiated into all embryonic lineages (Nichols and Smith, 2009). Likewise, murine trophoblast stem cells (mTSCs) can be isolated from blastocysts or early post-implantation embryos and can form all placental lineages (Tanaka et al., 1998). Unlike ESCs, which reflect a brief developmental window artificially perpetuated *in vitro*, mTSCs are actually present in mouse embryos. Their niche is a structure called the extra-embryonic ectoderm, which forms shortly after implantation and lacks a human counterpart (Maltepe and Fisher, 2015; Tanaka et al., 1998; Uy et al., 2002). Reflecting their cells of origin, mESCs and mTSCs are fixed in their specifications. In chimera assays, mESCs and mTSCs contribute only to

 © 2020 The Authors.
This is an open access article under the CC BY license (http://creativecommons.org/licenses/by/4.0/).

embryonic and placental lineage, respectively. mESCs cannot be converted to mTSCs *in vitro* except by genetic manipulation (Niwa et al., 2005), and even then the resulting cells are incompletely reprogrammed (Cambuli et al., 2014).

Finding human counterparts for these stem cells has proven to be more complicated, and their behavior has not always matched that of the murine counterpart. As with mice, human ESCs (hESCs) can be isolated from pre-implantation blastocysts (Thomson et al., 1998). However, in conventional medium (with serum and fibroblast growth factor 2 [FGF2]), hESCs have an epithelial morphology, high levels of DNA methylation, and a transcriptome resembling primed post-implantation epiblast (Nakamura et al., 2016; Nichols and Smith, 2009). Several formulations for culturing naive hESCs have been developed. Two formulations, 5iLAF (Theunissen et al., 2014) and t2iL + Gö (Takashima et al., 2014), show low DNA methylation and strong reactivation of pre-implantation genes, while other formulations show intermediate positions on the naive-primed spectrum (Pastor et al., 2016).

Efforts to obtain human trophoblast stem cells (hTSCs) from blastocysts using culture conditions analogous to murine TSCs have not been successful (Kunath et al., 2014). Primed hESCs treated with BMP4 and inhibitors of Activin and FGF signaling upregulate placental genes (Amita et al., 2013). However, the resulting cells differentiate and quickly stop dividing. Furthermore, there is argument as to whether they more closely resemble placenta or mesoderm (Roberts et al., 2014), with some evidence suggesting partial but incomplete reprogramming to a placenta-like state (Lee et al., 2016). Recently, hTSCs were successfully derived from first-trimester placental villi and pre-implantation blastocysts (Okae et al., 2018). Self-renewing placental organ cultures have also been derived from first-trimester placenta (Haider et al., 2018; Turco et al., 2018). hTSCs are clearly placental, have a very long or indefinite replicative life, and can differentiate to EVTs and STBs. hTSCs are epithelial cells and share key surface markers with villous CTBs. It remains unclear whether hTSCs are simply CTBs successfully adapted to *in vitro* culture or if they represent a CTB subpopulation or precursor.

Intriguingly, naive hESCs may reflect an earlier or less-fixed developmental state than mESCs. Their pattern of gene and transposon expression is especially primitive, corresponding to early epiblast or even late morula (Theunissen et al., 2016). Naive hESCs show some features typically associated with placental cells, including high TFAP2C levels (Pastor et al., 2018) and nuclear localization of YAP protein (Qin et al., 2016). A recent paper reported the existence of a subpopulation of cells in t2iL + Gö naive culture that had an expression pattern dissimilar from both naive and primed hESCs (Messmer et al., 2019). The identity of these cells was not determined, but they show upregulated expression of placental markers, such as *VGLL1*, *GATA2*, *GATA3*, and *XAGE3*, and are negative for the pluripotency markers *OCT4* and *NANOG*. Thus, even hESCs cultured in naive medium may undergo spontaneous differentiation to placental lineage.

We sought to determine whether naive hESCs can differentiate to the trophoblast lineage and form hTSCs. In addition to helping us understand the nature of naive human pluripotency, such a capability would allow generation of hTSC lines from existing hESC lines and could potentially be used to model human placental specification.

RESULTS

Similarity of hTSCs to Stem Cells in First-Trimester Placenta

To compare hTSCs and cells differentiated *in vitro* with primary placental cells, we conducted principal-component analysis (PCA) of published RNA-seq data from hTSCs and primary placental cell types (Okae et al., 2018). hTSCs clustered closer to CTBs than to differentiated EVTs and STBs, with *in-vitro*-differentiated cells positioned relatively close to their isolated *in vivo* counterparts (Figure S1A). Yet, there was still considerable distance between hTSCs and CTBs. While this may partially reflect adaptation to *in vitro* culture, we considered that hTSCs may represent a distinct subpopulation of CTBs.

A recent report described a subpopulation of proliferative cells at the base of the CTB cell column in first-trimester placental villi (Lee et al., 2018). These cells appear to give rise to EVTs and STBs and may be the core stem cell population in first-trimester placenta. These cells upregulate a number of genes relative to both CTBs and EVTs, and are distinguished by the surface markers ITGA2 and EpCAM (Lee et al., 2018). Interestingly, hTSCs express far higher levels of *ITGA2* and *EPCAM* than bulk CTBs (Figure 1A). Furthermore, genes identified as upregulated in these primary ITGA2$^+$ EpCAM$^+$ cells are expressed at globally higher levels in hTSCs than CTBs or differentiated placental cells (Figure 1B). We confirmed by flow cytometry that hTSCs are strongly positive for ITGA2 and EpCAM and downregulate expression of these genes upon differentiation (Figures 1C, 1D, and S1B). Together, these data suggestthat hTSCs may correspond to a real reported stem cell population in placenta and may explain some of the modest divergence observed between CTBs and hTSCs. Also, although neither marker is specific to placenta, ITGA2 and EpCAM may be used to sort hTSCs from heterogeneous populations.

Transdifferentiation of Naive hESCs to Putative hTSCs

To determine whether naive hESCs could be converted to placental lineage, we used an established reporter line

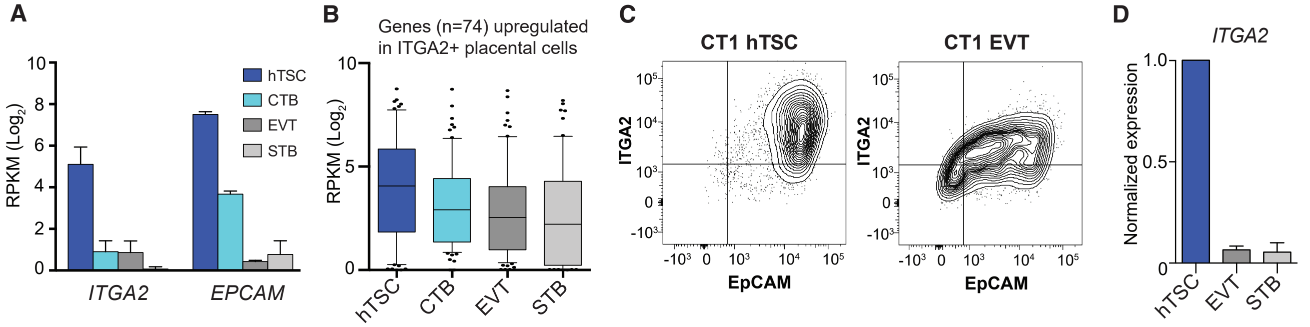

Figure 1. Similarity of hTSCs to reported ITGA2$^+$ EpCAM$^+$ Progenitor Population

(A) Expression of *ITGA2* and *EPCAM* in hTSCs and primary placenta cells. Data are taken from Okae et al. (2018), with n = 3–4 independent experiments per cell type.

(B) Seventy-four genes were identified as upregulated in ITGA2$^+$ cells by Lee et al. (2018) and also present in Okae et al.'s RNA-seq dataset. The expression of these genes is plotted using RNA-seq data from Okae et al., with each gene represented as a single point in the boxplot.

(C) Flow cytometry plot of ITGA2 and EpCAM in CT1 hTSCs and EVT. Representative of n = 5 independent experiments.

(D) Downregulation of ITGA2 upon directed differentiation of CT1 (qRT-PCR, mean + SE of n = 2 independent experiments).

(WIBR3 OCT4-ΔPE-GFP) that expresses GFP only upon acquisition of naive pluripotency (Theunissen et al., 2014). We treated the cells with a rapid naive induction protocol that entails culture with PXGL media (Guo et al., 2017), an improved version of the well-established t2iL + Gö naive media (Takashima et al., 2014). We observed GFP$^+$ colonies with dome-shaped naive morphology (Figures 2A and 2B) and strong upregulation of naive markers (Figure S2A). Ten days after the start of reversion, we plated the hESCs directly into hTSC medium. Although a mixed population of cells formed, within 4 days of transition we observed colonies of epithelial cells that resembled hTSCs (Figure 2C) and stained strongly positive for the pan-placental markers KRT7 and TFAP2C and the CTB marker TEAD4 (Figure 2D). After 11 days of culture in hTSC medium, we sorted a pure population of putative hTSCs using the surface marker profile ITGA2^{hi} EpCAM^{hi} ITGA1^{lo} (Figure 2E). ITGA2 and EpCAM were chosen on the basis of the observations above, and ITGA1 was selected against because it is expressed on differentiated placental cells (Nagamatsu et al., 2004) but is low in hTSCs (Figures 2E and S2B). We also stained for HLA-G to gate against differentiated HLA-G^{hi} cells, although we eventually ceased use of this marker because it was less sensitive than ITGA1. The resulting transdifferentiated hTSC (tdhTSC) line (termed WIBR3-tdhTSC line 1) was morphologically indistinguishable from hTSCs of placental origin (Figure 2F) and had similar surface marker expression (Figure S2C). To rule out the possibility of contamination with placental hTSCs, we conducted short tandem repeat (STR) analysis and confirmed that WIBR3-tdhTSC L1 has the same genetic markers as the starting WIBR3 hESCs (Table S1). A list of all transdifferentiations conducted and cell lines generated in this paper are included in Table S2.

As discussed in the introduction, there is evidence that a very small proportion of cells in t2iL + Gö steady-state culture may already express placental markers, and it is possible that some cells in naive culture conditions have not fully attained naive state. To establish that genuine naive hESCs, rather than a side population, are what give rise to tdhTSCs, we sorted GFP^{hi} and GFP^{lo} WIBR3 OCT4-ΔPE-GFP naive-cultured cells into hTSC medium (Figure 2G). The GFP^{hi} hESCs (true naive) gave rise to ITGA2$^+$ EpCAM$^+$ cells with far higher efficiency, as demonstrated by fluorescence-activated cell sorting (FACS) 16 days later (Figure 2H). ITGA2^{hi} EpCAM^{hi} ITGA1^{lo} cells were sorted to give rise to an additional line, WIBR3 tdhTSC line 2 (Figures 2H and S2D). The GFP^{lo} hESCs by contrast gave rise to very few ITGA2$^+$ EpCAM$^+$ cells (Figure 2H).

We also reverted and transdifferentiated a second embryonic stem cell line, UCLA1 (Diaz Perez et al., 2012), via the same strategy (Figures S2E and S2F). All lines generated showed uniform staining for KRT7, TFAP2C, and TEAD4 (Figure 2I).

Validation of Putative Transdifferentiated hTSCs

To confirm placenta-like identity of the putative tdhTSCs, we conducted RNA-seq of the starting hESC lines, tdhTSCs, and control placental (CT1, CT3) and blastocyst-derived (BT2) hTSCs. As a further comparison, we sequenced RNA from two epithelial cell lines: FT190-transformed fallopian tube epithelium and Hec116 endometrial carcinoma. A full list of samples and mapping statistics is in Table S3, with gene expression levels in Table S4.

Appropriately, tdhTSCs show dramatically reduced expression of core pluripotency transcription factors and gain of established placental markers and CTB/hTSC genes (Figures 3A and 3B). Minimal expression of amnion or

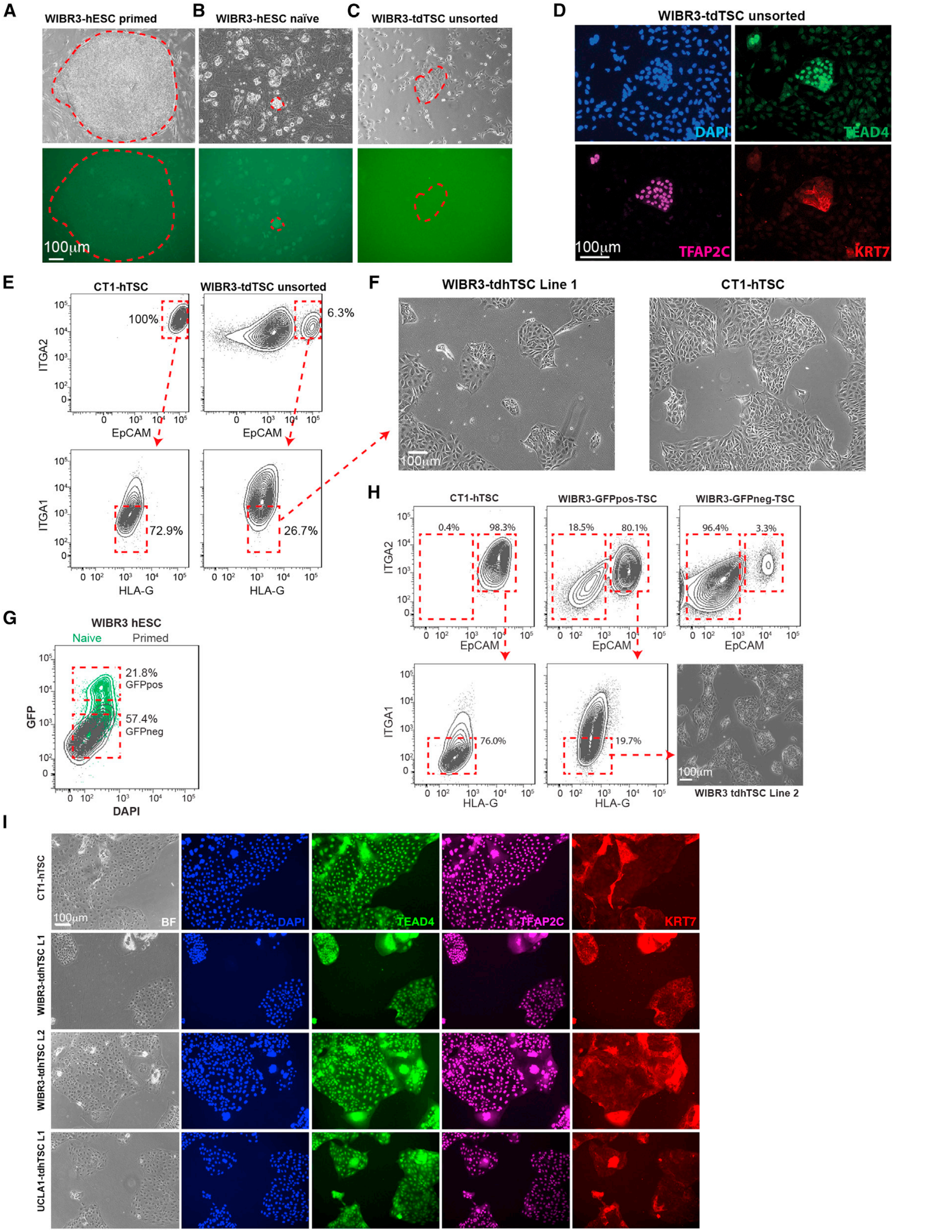

(legend on next page)

somatic differentiation markers was observed. PCA of gene expression data showed three clear clusters: hESCs, hTSCs, and tdhTSCs, and non-placental epithelial lines (Figure 3C), with tdhTSCs intermingled with genuine hTSCs.

Classifying cells as placental on the basis of expression of a small number of markers is controversial (Roberts et al., 2014). Classic pan-placental markers, such as TFAP2C, GATA3, and KRT7 are expressed in many non-placental tissues, as are the hTSC/CTB genes ELF5, TP63, and TEAD4 (Uhlen et al., 2015). Even the pregnancy hormone hCG is produced in pituitary cells (Chen et al., 1976). Therefore, we used a non-biased approach to identify genes expected to show increased expression in placenta. We analyzed gene expression data from early primate embryogenesis (Nakamura et al., 2016) and identified 107 genes specific to trophoblast as compared with epiblast, hypoblast, and gastrulating cells, 89 of which were expressed (reads per kilobase of transcript per million mapped reads [RPKM] > 1) in at least one of our RNA-seq samples (Table S5). As predicted, hTSCs show dramatically higher expression of these trophoblast-specific genes than do pluripotent or epithelial cells (Figure 3D). Crucially, tdhTSC lines express trophoblast genes at levels similar to hTSCs of placental origin.

Moreover, the tdhTSCs show other classic hallmarks of placental identity (Lee et al., 2016). They exhibit high expression of microRNAs generated from the Chromosome 19 microRNA cluster (C19MC) (Figure S3A), demethylation of the *ELF5* promoter (Figure S3B), and reduced staining with pan-HLA antibody relative to non-placental epithelial cell lines (Figure S3C). They were also capable of directed differentiation to STB lineage, upregulating STB markers and secreting large quantities of hCG (Figures 4A, 4B, S4A, and S4C). Differentiation of WIBR3 tdhTSCs to EVT lineage resulted in spindle-shaped morphology, gain of the EVT markers ITGA1 and HLA-G, and upregulation of EVT genes (Figures 4A, 4C, S4B, and S4C). UCLA1 tdhTSC L1 show impaired differentiation to mature EVTs (Figure 4A and data not shown), although so do some blastocyst-derived hTSC lines (Okae et al., 2018).

Combined, these results demonstrate similarity of tdhTSCs to genuine hTSCs.

Gain of Placenta-like DNA Methylation Pattern in tdhTSCs

The placenta has a highly distinctive DNA methylation profile compared with hESCs or somatic cells. Global methylation levels are much lower in placenta and especially low in hTSCs (Okae et al., 2018). Despite this reduced overall DNA methylation level, some CpG islands have increased DNA methylation in placenta and hTSCs, a pattern that is often recapitulated in the CpG island methylator phenotype, which occurs in many somatic cancers (Smith et al., 2017). We sought to determine whether tdhTSCs acquired this distinctive placenta-like methylome. Also, because DNA methylation is a highly heritable mark, we reasoned that even if tdhTSCs have acquired an overall hTSC-like phenotype, they might still bear traces of their cell of origin or their period as naive hESCs.

We performed whole-genome bisulfite sequencing of CT1, CT3, WIBR3 primed hESCs, and WIBR3 tdhTSC line 1 and line 2 (Table S3). Methylation levels and patterns observed for CT1 and WIBR3 hESCs are similar to published data (Figures 5A and S5A). The global CpG DNA methylation level of the tdhTSCs is similar to that of placental hTSCs and far lower than that of hESCs (Figure 5A). The methylation level of individual CpG islands and gene promoters in tdhTSCs is far better correlated with that of placental hTSCs than with hESCs (Figures 5B, 5C, and S5B). On a global level, tdhTSCs appear to acquire a placenta-like methylome.

We then focused on the phenomenon of placenta-specific CpG island methylation. We used published data to identify 788 CpG islands with substantially higher methylation in CT1 hTSCs compared with WIBR3 hESCs

Figure 2. Transdifferentiation of hESCs to Putative hTSCs and Purification via FACS Sorting

(A) Upper panel: light microscopy image of a colony of primed WIBR3 OCT4-ΔPE-GFP hESCs, with the colony circled with a dashed line. Lower panel: lack of GFP signal.

(B) Light and fluorescent image of WIBR3 OCT4-ΔPE-GFP hESCs after 10 days of naive reversion. Many GFP^+ colonies are present, with one representative colony circled.

(C) Naive hESCs after 4 days of culture in hTSC medium. Note the presence of a colony of epithelial cells and the loss of GFP signal.

(D) Immunofluorescent image of WIBR hESCs after 10 days in hTSC medium. Note a distinct population of $TFAP2C^+$ $TEAD4^+$ $KRT7^+$ cells.

(E) FACS of WIBR3 hESCs grown in hTSC medium for 11 days and comparison CT1 hTSCs. Note distinct population of $ITGA2^{hi}$ $EpCAM^{hi}$ cells which was sorted to produce WIBR3 tdhTSC line 1.

(F) Light microscopy of CT1 and WIBR3 tdhTSC line 1.

(G) Flow cytometry of WIBR3 OCT4-ΔPE-GFP hESCs in naive (green) and primed (black) conditions are overlaid. GFP^{hi} and GFP^{lo} cells from naive culture, populations indicated with boxes, were sorted into hTSC medium.

(H) Flow cytometry of GFP^{hi} and GFP^{lo} cells after 16 days in hTSC medium. Note much higher $EpCAM^{hi}$ $ITGA2^{hi}$ population in the GFP^{hi} population.

(I) Immunofluorescent staining for hTSC/CTB (TEAD4) and pan-placental (TFAP2C, KRT7) markers in lines indicated. Representative of n = 2 independent experiments.

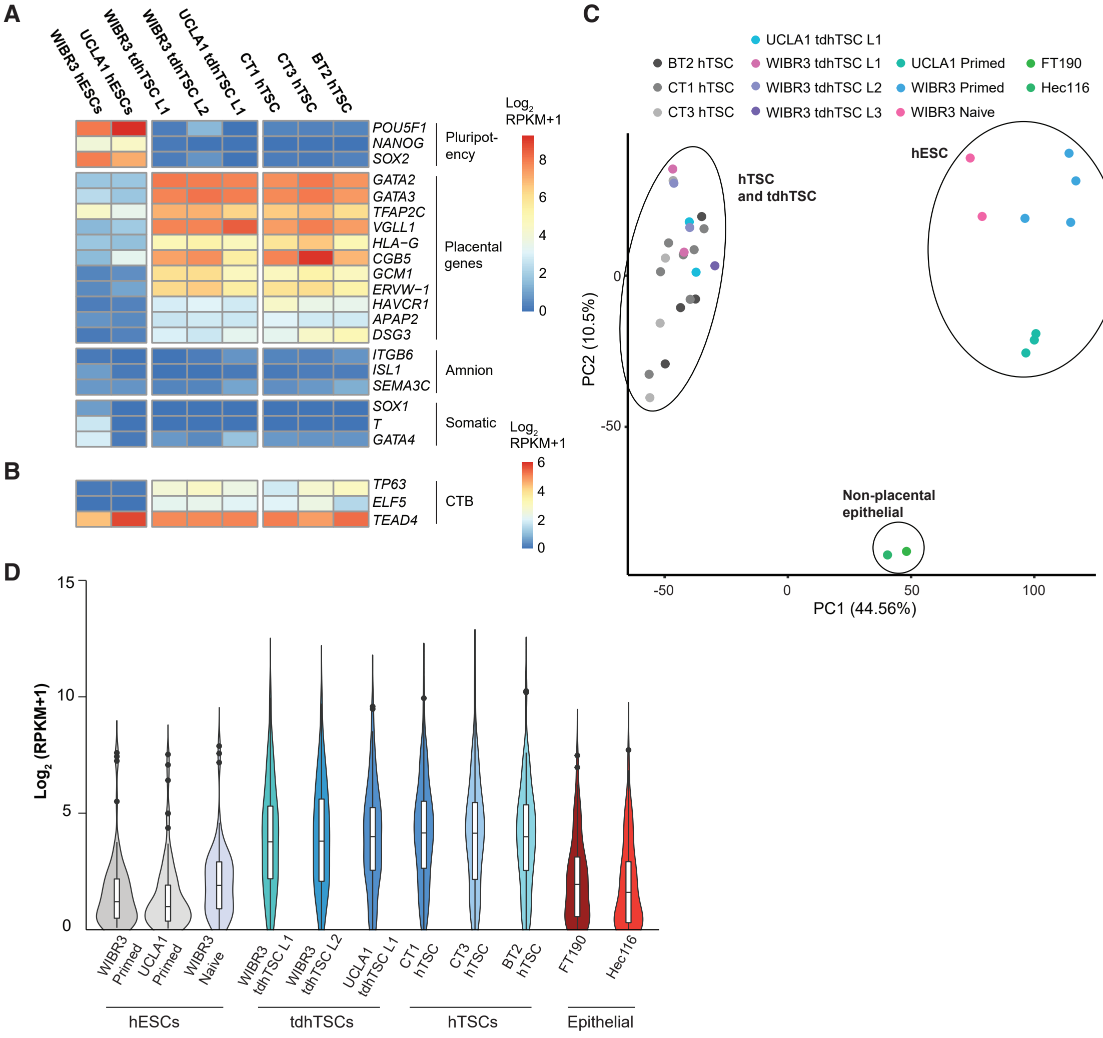

Figure 3. Validation that Transdifferentiated hTSCs Express Placental Genes

(A) Gene expression of indicated markers in each sample type are indicated by coloration. Expression from replicates of each sample type are averaged. n = 2 (all tdhTSC lines), n = 3 (UCLA1 hESC), n = 4 (WIBR3 hESCs, CT3, BT2), n = 7 (CT1) biological replicates.

(B) Same as (A) except with a different color scale.

(C) Principal-component analysis for gene expression of the lines indicated. Each dot is one biological replicate.

(D) Expression of 89 trophoblast-specific genes, as identified by analysis of pre-implantation primate embryos (Nakamura et al., 2016), is indicated for each cell type. Expression of each gene, using an average of all replicates for a given cell type, is indicated as a single point on the violin plot. Box indicates 25th, 50th, and 75th percentiles. n = 1 (epithelial cell), n = 2 (all tdhTSC lines, naive hESCs), n = 3 (UCLA1 hESCs), n = 4 (WIBR3 hESCs, CT3, BT2), n = 7 (CT1) biological replicates.

(see Experimental Procedures and Table S6). Interestingly, these CpG islands include the promoters of genes critical for neural lineage (*SOX1*, *PAX6*), cardiac development (*HAND2*, *NKX-2.5*), and transforming growth factor β family signaling (*NODAL*, *FOXH1*), suggesting that DNA methylation may be a mechanism for shutting off these lineages in placenta. The WIBR3 tdhTSCs show a strong increase in DNA methylation at these 788 CpG islands relative to primed cells (Figures 5D and 5E), demonstrating that tdhTSCs have gained placenta-specific methylation.

We next considered the phenomenon of "gatekeeper" genes: key placental genes whose promoters are methylated in hESCs, thus precluding conversion to placental fate. Analogous methylation is a critical obstacle to complete conversion of murine ESCs to mTSCs (Cambuli et al., 2014). To address this question, we identified 2,107 promoters methylated in WIBR3 hESCs that show dramatically lower DNA methylation in CT1 hTSCs (see Experimental Procedures and Table S6). These regions show dramatic loss of methylation in WIBR3 tdhTSCs

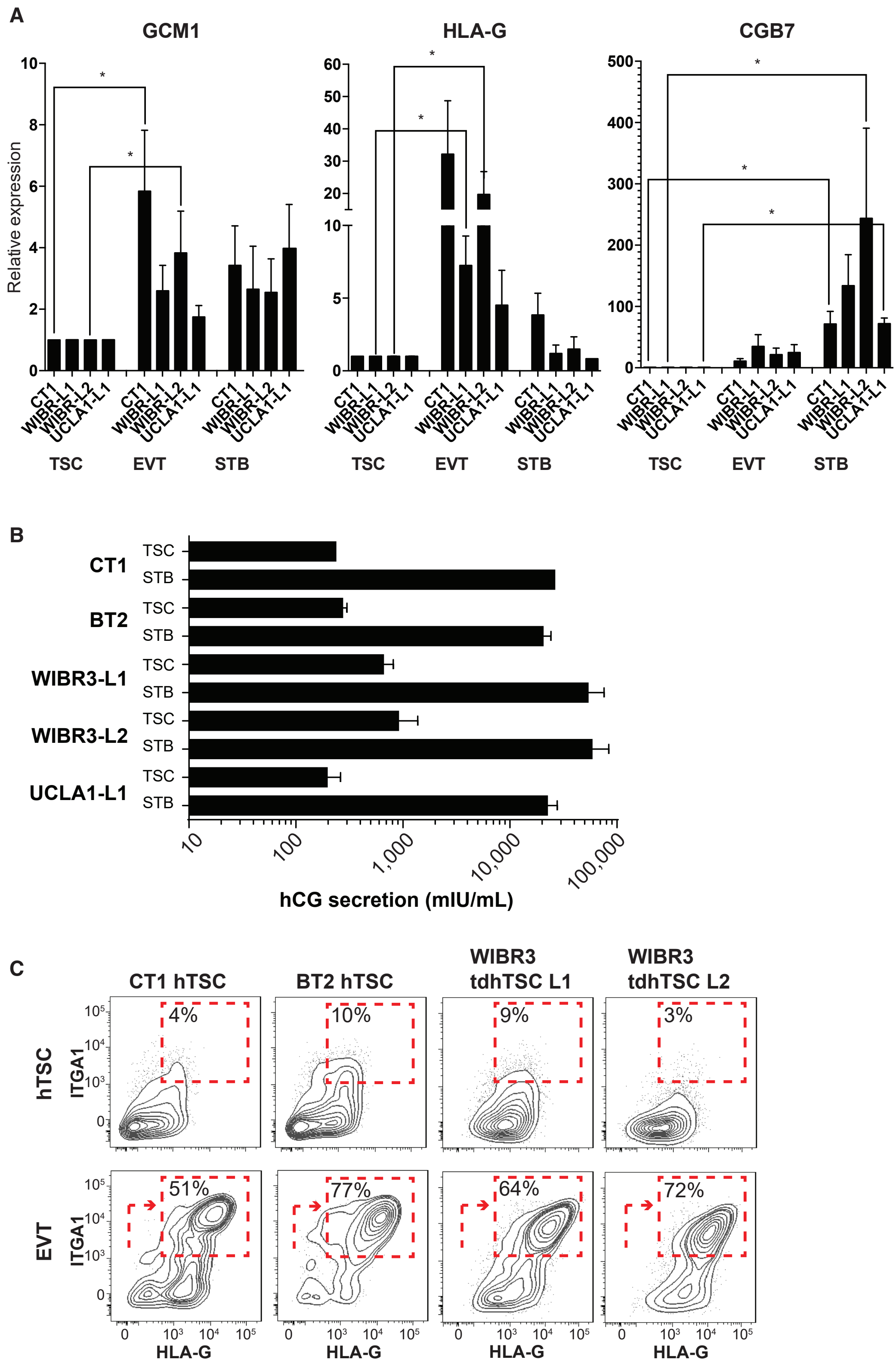

(legend on next page)

(Figure 5F). Of these genes, 172 are upregulated in hTSCs relative to primed hESCs and have an average RPKM > 1 in hTSCs, possible gatekeepers (Table S6). This set includes key placental factors, including *ELF5* and a number of hCG and STB fusion genes. Globally, these genes show increased expression in tdhTSC lines, comparable with placental hTSCs (Figure 5G). There is thus no general inability to reactivate placental gene expression in tdhTSCs, even at genes whose promoters are heavily methylated in starting hESCs.

Dysregulation of Select Imprinted Genes in tdhTSCs

Nonetheless, we detected examples of aberrant methylation in tdhTSCs in which their past as naive or primed hESCs was apparent. As expected, many imprinted regions showed aberrant hypomethylation in hTSCs (Figure 6A), a predictable consequence of having once been naive hESCs (Pastor et al., 2016). More surprisingly, three imprints showed selective hypermethylation in tdhTSC: *PEG3*, *ZFAT*, and *PROSER2-AS1*. *PEG3* is low expressed in primed hESCs and is prone to hypermethylation in culture, while *ZFAT* and *PROSER2-AS1* are placental imprints that converge toward methylation in pluripotent and somatic cells (Barbaux et al., 2012; Hamada et al., 2016). Apparently, they resisted demethylation in both the naive and trophoblastic states.

We calculated differentially expressed genes, comparing hTSCs with both primed hESCs and tdhTSCs (Figures 6B and 6C; Table S7). Very few genes show substantial dysregulation in tdhTSCs relative to hTSCs, but they include *PEG3*, *ZFAT*, and *PROSER2-AS1*, all of which show striking downregulation consistent with their hypermethylation (Figures 6C and S6). In summary, while tdhTSCs have a broadly hTSC-like methylome, they do carry vestiges of their past that have some impact on their transcriptional program.

Conversion of Non-naïve Cells to hTSC-like Cells

A recent report details the generation of "expanded potential stem cells" (EPS cells). EPS cells have a transcriptional program and level of DNA methylation similar to primed hESCs but can reportedly be differentiated into trophoblast by treatment with BMP4. When cultured in hTSC medium, trophoblast-like colonies can be identified, picked, and propagated (Gao et al., 2019). A second report countered the first claim, showing that EPS cells treated with BMP4 formed cells with amnion-like, rather than trophoblast-like, properties (Guo et al., 2020). However, it remained unclear whether tdhTSCs could be derived from EPS hESCs, so we analyzed RNA-seq data from the EPS-derived tdhTSCs. Although they were not sequenced in parallel with placental hTSCs, making direct comparison difficult, they express trophoblast genes at levels similar to placental hTSCs (Figure S7A) and do not express amnion-specific genes at high levels (Figure S7B). Interestingly, two of the three tdhTSC lines made from EPS cells showed low *PEG3* expression, and all showed low *ZFAT* and *PROSER2-AS1* expression (Figure S6).

To perform a direct comparison, we grew three hESC lines (WIBR3, UCLA1, and H9) in naive medias (5iLAF, PXGL), in medias that produce cells with a mixture of naive and primed properties (EPS, RSet), and in primed medium, and subsequently transferred them to hTSC medium for 15 days. Surprisingly, all conditions were able to produce at least some population of ITGA2^{hi} EpCAM^{hi} ITGA1^{lo} cells (Figures 7A and S7C; Table S2). However, naive conditions generally produced more efficient transdifferentiation as measured by production of ITGA2^{hi} EpCAM^{hi} ITGA1^{lo} cells (Figure S7C). WIBR3 cells had markedly inefficient transdifferentiation from EPS medium (Figure S7C), to the extent that we were not able to isolate a pure line. Furthermore, while tdhTSC lines isolated from naive cells showed uniformly low staining for the amnion marker ITGB6 (Guo et al., 2020), lines generated from EPS, RSet, or primed UCLA1 or H9 cells showed heterogeneous staining for this mark and higher expression as measured by RT-PCR (Figures 7B–7D). Expression of the gene *HAVCR1*, specific to trophoblast over amnion (Guo et al., 2020), was uniformly higher in tdhTSCs generated from naive cells (Figure 7D). Finally, while naive hESCs gave rise to uniformly TEAD4^{+} TFAP2C^{+} KRT7^{+} tdhTSC colonies after sorting, EPS, RSeT, and primed-derived tdhTSCs contained a second population of cells with markedly lower TEAD4 staining (Figure S7D). It remains unclear whether EPS, RSet, and primed cells gave rise to a mixture of hTSCs and amnion-like cells, or cells with properties of both, but naive cells are clearly optimal for generation of hTSCs.

Figure 4. Differentiation Capacity of tdhTSCs

(A) qRT-PCR for markers of EVT (*GCM1*, *HLA-G*), and STB (*GCM1*, *CGB7*) differentiation of lines indicated, normalized to *GAPDH*. Error bars indicate mean + SE for n = 3–5 independent experiments. *p < 0.05 in one-tailed t test.

(B) ELISA assay for hCG secretion after directed differentiation to STB. Error bars indicate mean + SE for n = 2 biological replicates, except CT1 for which there is one replicate.

(C) Flow cytometry of hTSCs and EVT, for the EVT markers HLA-G and ITGA1, for the lines indicated. Representative of n = 5 independent experiments.

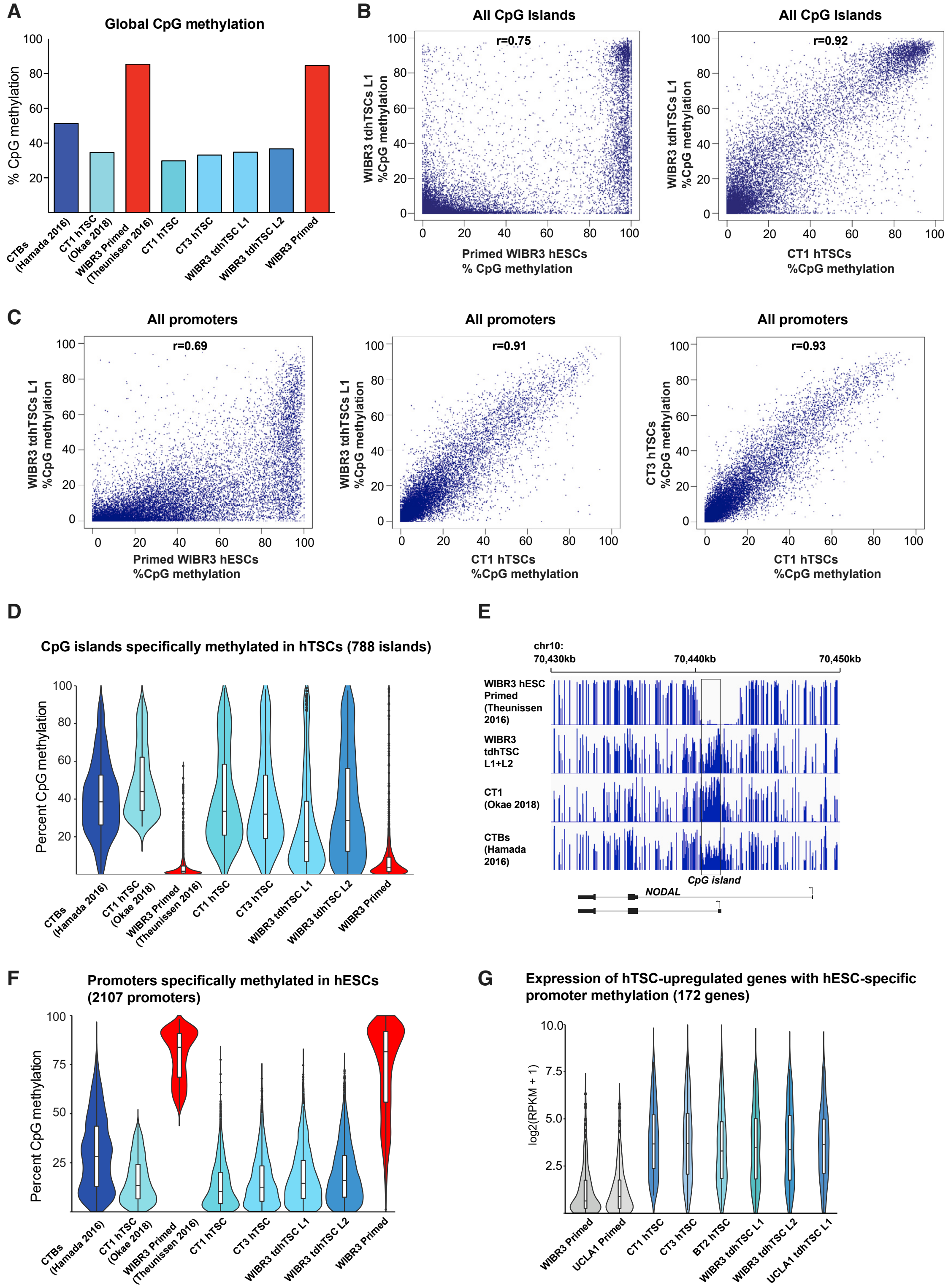

Figure 5. Global Methylation Patterns of tdhTSC

For all data, data in parentheses indicate data mined from published sources, data without parentheses indicate original data.

(A) Global CpG methylation level in samples indicated.

(legend continued on next page)

DISCUSSION

We find that naive hESCs can be converted into hTSC-like cells. Very recently, another report also demonstrated production of hTSC-like cells from naive hESCs (Dong et al., 2020). Whereas we cultured naive cells in hTSC medium for 10–22 days and purified tdhTSCs using surface markers, Dong and colleagues cultured naive cells in hTSC medium for 5–10 passages and report pure tdhTSC lines. There is an unproven but plausible reconciliation of these findings: successfully transdifferentiated tdhTSCs may grow faster than other cell types in mixed transdifferentiations, and may dislodge other cells as tdhTSC colonies expand, resulting in their taking over mixed cultures even without sorting.

Imprinting in tdhTSCs

It is probably impossible to generate perfectly placenta-like tdhTSCs from hESCs by any current method. Certain parental imprints are retained only in placenta and accordingly these regions show biallelic hyper- or hypomethylation in hESCs (Okae et al., 2014). hESC lines frequently show aberrations even in non-placental imprints (Rugg-Gunn et al., 2007), and culture in naive conditions results in widespread imprint erasure (Pastor et al., 2016; Theunissen et al., 2016). Considering the importance of imprinting in placental development, it is somewhat remarkable that tdhTSCs are as similar to hTSCs as they are. Human conceptuses that lack maternal imprinting give rise to hydatidiform moles, aberrant placentas with little or no embryonic tissue (Nguyen and Slim, 2014). Nonetheless, only three imprinted genes failed to activate upon transdifferentiation. The failure to reactivate *PEG3* is somewhat surprising, because this imprint is demethylated and chromatin-opened in naive hESCs (Pastor et al., 2016). This may be a consequence of the relatively brief period in which the hESCs used in this study were cultured in naive medium. *ZFAT* and *PROSER2-AS1* by contrast do not show open chromatin in naive hESCs (Pastor et al., 2016), are not reactivated in tdhTSCs derived from EPS cells, and may be the most refractory to activation in tdhTSCs.

More surprising is two imprints which were not differentially expressed: *IGF2* and *CDKN1C*. Demethylation of the H19 and KvDMR1 loci, respectively, should be expected to abrogate long-range promoter-enhancer interactions necessary for the expression of these two genes (Soejima and Higashimoto, 2013). *CDKN1C* is of particular note because it is strongly implicated in molar pathology, and its loss eliminates contact inhibition in hTSCs (Takahashi et al., 2019). However, expression of these genes was highly variable across both hTSC and tdhTSC replicates and trended lower in tdhTSCs (Table S3).

Transdifferentiation Capacity of Human Pluripotent Cells

While we show that naive hESCs are more efficient in transdifferentiating to tdhTSCs, it remains unclear whether hTSC-like cells can be generated from non-naive cells or how such cells might be different or impaired. There are now reports of generation of trophoblast-like cells from EPS cells (Gao et al., 2019), primed hESCs treated with BMP4 and an S1P3 agonist (Mischler et al., 2019), and primed hESCs cultured in micromesh (Li et al., 2019). Our results certainly do not rule out this possibility. However, distinguishing tdhTSCs from contaminating, possibly amnion-like cells, is not a trivial endeavor: the contaminating cells share surface markers (ITGA2, ITGA6, and EpCAM) and show only subtle morphological differences from hTSCs. Amnion expresses many of the same core transcription factors as placenta (Guo et al., 2020). Amnion identity must be firmly ruled out, preferably by direct comparison with placental hTSCs.

While hESCs can convert to tdhTSCs, naive murine ESCs require genetic manipulation to make such a transition and even then do so incompletely (Cambuli et al., 2014). There are at least two possible explanations, which are not mutually exclusive: human naive pluripotent cells may reflect an earlier developmental state than murine naive cells or human pluripotent cells may retain greater plasticity than

(B) Correlation of CpG island methylation in each of the two samples is indicated. Each CpG island represents as a single point, all CpG islands with adequate coverage are plotted.
(C) Correlation of promoter methylation between two samples is indicated. All autosomal promoters with adequate coverage are plotted.
(D) Violin plot indicating degree of CpG island methylation in samples indicated among 788 CpG islands that show higher DNA methylation in CT1 hTSCs relative to primed hESCs.
(E) DNA methylation of a region of genome that includes the CpG island promoter of *NODAL*. Height of bars corresponds to percentage CpG methylation, from 0% to 100%. Data from WIBR3 tdhTSC L1 and L2 are merged to allow sufficient sequencing depth for visualization.
(F) Violin plot showing methylation level of 2,107 promoters that show higher DNA methylation in primed hESCs relative to CT1 hTSCs.
(G) Expression of 172 possible "gatekeeper" genes, genes that have upregulated expression in hTSCs and higher promoter methylation in hESCs. Expression of each gene, using an average of all replicates for a given cell type, is indicated as a single point on the violin plot. Box indicates 25th, 50th, and 75th percentiles. n = 2 (all tdhTSC lines), n = 3 (UCLA1 hESCs), n = 4 (WIBR3 hESCs, CT3, BT2), n = 7 (CT1) biological replicates.

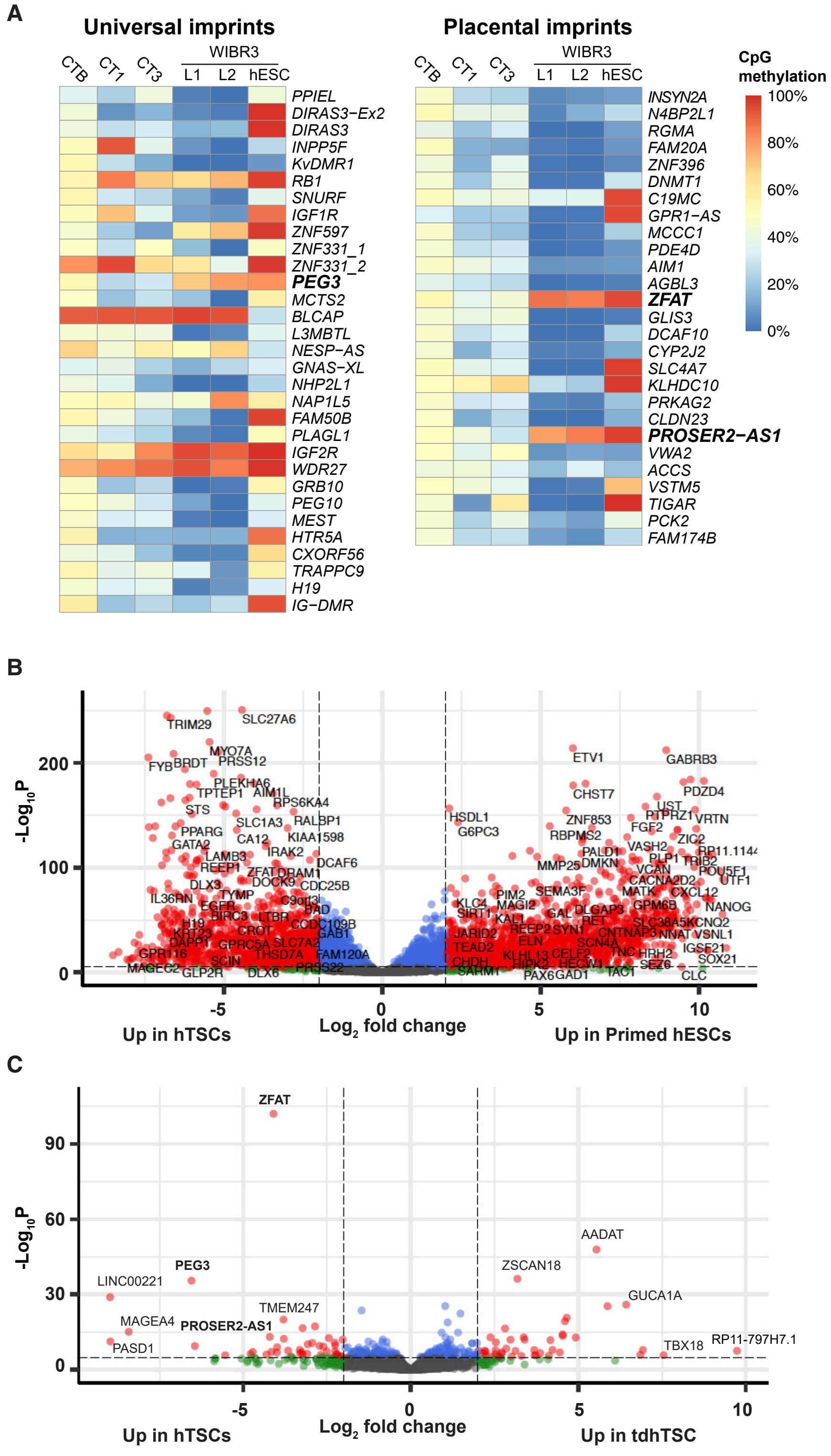

Figure 6. Imprinting Abnormalities in tdhTSC

(A) CpG methylation level of all universal (organism-wide) and placental imprints in cell types indicated. CTB, primary cytotrophoblasts; L1, WIBR3 tdhTSC L1; L2, WIBR3 tdhTSC L2. CTB data from Hamada et al. (2016), original data shown for all other samples.

(B) Volcano plot of genes differentially expressed in hTSCs (CT1, CT3, and BT2) versus primed (WIBR3 and UCLA1 hESCs). Red dots correspond to differentially expressed genes ($p_{adj} < 0.05$, fold-change > 4).

(C) Volcano plot of genes differentially expressed in hTSCs (CT1, CT3, and BT2) versus tdhTSCs (WIBR3 tdhTSC lines 1, 2, and 3, and UCLA1 line 1). Red dots correspond to differentially expressed genes ($p_{adj} < 0.05$, fold-change > 4).

previously appreciated. The latter phenomenon deserves serious consideration. Remarkably, cells from day 5 human blastocysts (stage BL3), if harvested and reaggregated into an empty zona pellucida, will compact and cavitate, forming blastocysts of normal appearance with a NANOG^{+} ICM (De Paepe et al., 2013). Similar results are obtained if only

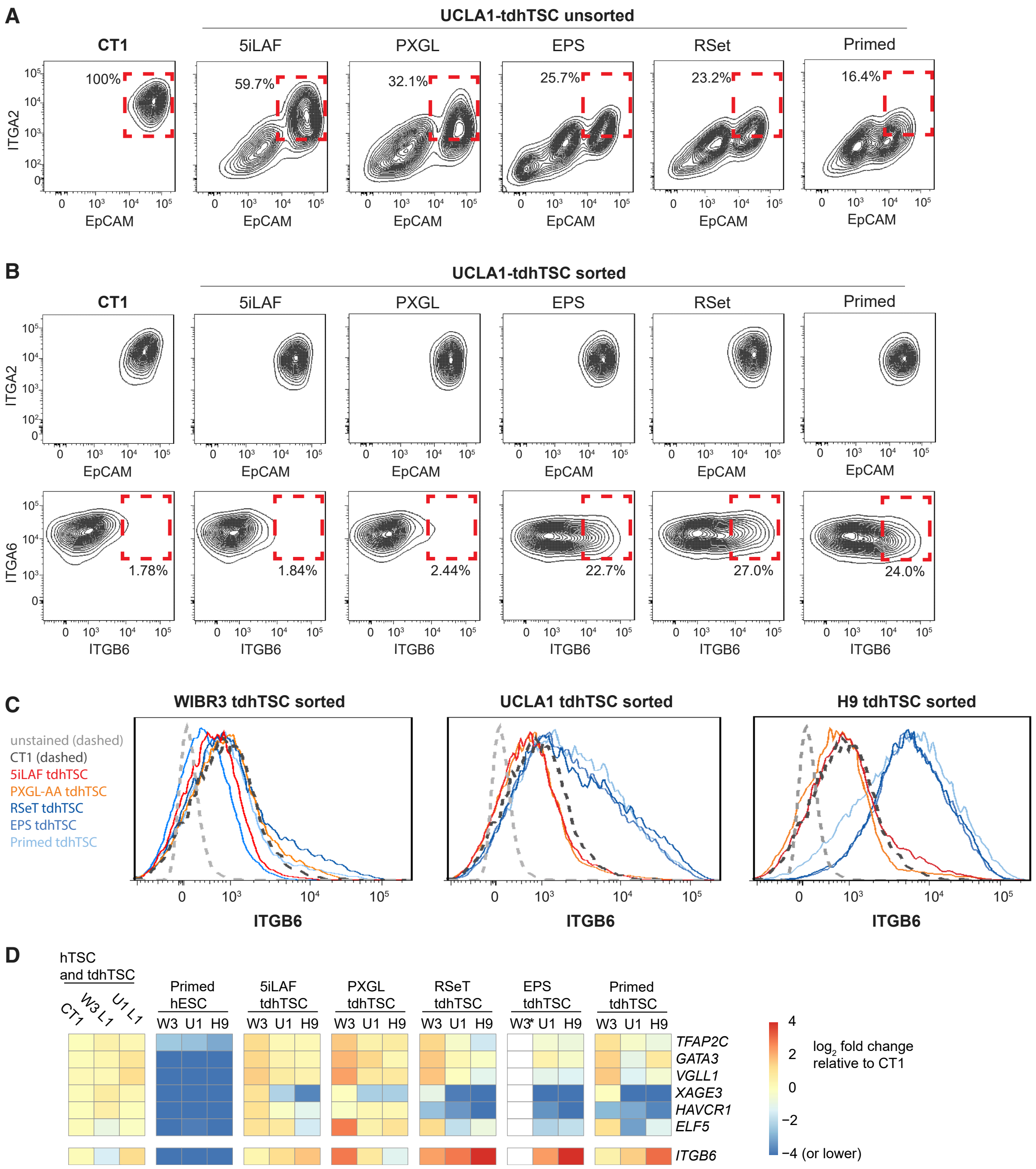

Figure 7. Comparative Transdifferentiation Capacity from Different Media Conditions

(A) Flow cytometry of UCLA1 hESCs cultured in various conditions and then grown in hTSC medium for 15 days. Percentage of ITGA2^{hi} EpCAM^{hi} cells is indicated.

(B) Flow cytometry 20 days after sorting. Note higher ITGB6 signal in putative tdhTSCs derived from hESCs in non-naive conditions.

(C) Flow cytometry histogram of ITGB6 signal for putative tdhTSCs derived from hESC lines, and growth condition indicated.

(D) qRT-PCR quantification of placental markers and *ITGB6* in indicated cell lines normalized to *GAPDH*. W3, WIBR3; U1, UCLA1. *WIBR3 EPS-derived tdhTSCs could not be studied because conversion efficiency was too low to produce a pure ITGA2^{hi} EpCAM^{hi} line. n = 1 biological replicate.

outer (trophectoderm) or inner (ICM) cells are added to an empty zona pellucida, implying that lineage is not restricted until well after blastocyst formation. Furthermore, the barrier to placental transdifferentiation is sometimes crossed in the context of malignancy: early germ cells, which reactivate much of the transcriptional program

of the pluripotent epiblast, can give rise to choriocarcinomas (trophoblast-like tumors) in both mice and humans (Alison et al., 1987; Rijlaarsdam et al., 2015). Future studies may also indicate when and how in human development a firm barrier to placental differentiation is established.

EXPERIMENTAL PROCEDURES

TSC Culture and Differentiation

hTSCs were cultured according to published protocol (Okae et al., 2018) with the following alterations. 0.5–1 × 10^5 cells were plated on each well of a 6-well plate, and cells were passaged every 5–7 days. We observed that reduced oxygen levels promote hTSC self-renewal but inhibit directed differentiation, so we cultured hTSCs in 5% O_2 5% CO_2 but performed differentiation to EVT or STB at 20% O_2 5% CO_2.

EVT and STB differentiation were performed according to published protocol, with the following alterations. For STB differentiation the density of the cells when plated was doubled to 150,000 per 6-well plate well and cells were cultured for 3 days in STB (2D) medium before collection and assessment. For EVT, 150,000 cells were plated initially, but procedures for differentiation remained unchanged.

The three lines described in this paper as CT1, CT3, and BT2 correspond to the published lines TS^{CT1}, TS^{CT3}, and TS^{BLAST2}, respectively(Okae et al., 2018).

Embryonic Stem Cell Culture

Primed hESCs were routinely cultured with TeSR-E8 (StemCell Technologies) on hESC Qualified Matrigel (Corning). hESCs were reverted to naive state using a one-step induction adapted from published protocols (Guo et al., 2017). Further details are provided in Supplemental Experimental Procedures.

For consistency, all stem cells used in RNA-seq were cultured in 5% O_2.

Transdifferentiation of Naive hESCs to hTSC Culture

Naive hESCs were passaged to Matrigel (Corning)-coated plates at 20%–30% confluency into TSC culture medium. Cells were grown on collagen-coated plates in subsequent passages, akin to control hTSCs. Cells were grown to confluency before fluorescent cell sorting, which is described further in Supplemental Experimental Procedures.

STR Analysis

STR analysis was performed at the SickKids Center for Applied Genomics Facility using GenePrint10 (Promega).

hCG ELISA

hCG secretion was measured using an hCG AccuBind ELISA (Monobind) according to manufacturer instructions.

Generation of RNA-Seq Libraries

RNA extraction was performed using QIAGEN RNeasy Micro Kit, except for two samples (WIBR3 primed replicate 1, WIBR3 naive day 10) that were extracted using RNAzol RT (Sigma). RNA quality was confirmed using Bioanalyzer. mRNA was enriched from 500 ng total RNA using NEBNext Poly(A) mRNA Magnetic Isolation Module Kit and libraries were generated using Swift RNA Library Kit.

Samples were run on an Illumina NovaSeq instrument at the La Jolla Institute for Allergy and Immunology Sequencing Core, or on a HiSeq 4000 at Michael Smith Genome Sciences Center. Three samples were sequenced at both locations to confirm similarity of results.

RNA-Seq Analysis

Mapping and RPKM Calculation

FASTQ files were mapped to hg19 using the STAR aligner (v2.5.3a) with default parameters. Bam files were analyzed by RNA-SeQC to confirm library quality. Read counts were calculated with htseq-count and RPKM was calculated using cufflinks (v2.2.1) with default settings.

PCA

PCA was performed using prcomp function in R and plotted with the ggfortify package. Genes with RPKM < 2 in all samples were excluded from analysis.

Identification of Trophoblast-Specific Genes

Cynomolgus monkey single-cell RNA-seq data normalized using the RPM method were obtained from Nakamura et al. (2016). Trophoblast-specific genes were identified by calculating differentially expressed genes between trophoblast cells (11 cells each in the categories “Pre-implantation Early trophoblast,” “Pre-implantation late trophoblast,” and “Post-implantation parietal trophoblast”) and all other cells. Differentially expressed genes were identified as false discovery rate (FDR) < 0.05 using the kruskal.test function and p.adjust function in R. As a further filter, trophoblast-specific genes were required to show at least 4-fold higher expression in placental cells over all non-placental cell types and to have an average RPM > 7 in placental cells.

Differential Gene Expression Calling and Volcano Plot

Read counts obtained from htseq-count were used for differential gene expression with DESeq2. Genes with raw read count >100 were plotted using EnhancedVolcano package in R.

Generation of Whole-Genome Bisulfite Sequencing Libraries

Genomic DNA was collected using a QIAGEN Blood and Tissue Kit, including RNase A treatment. DNA concentration was measured using Nanodrop. DNA (500 ng) was fragmented using a Covaris M220 instrument and 250 ng was processed with a Zymogen bisulfite conversion kit. The equivalent of 50–100 ng of DNA was used to generate the sequencing library with the Accel-NGS Methyl-Seq DNA Library Kit (Swift Biosciences).

Libraries were sequenced as 150-bp paired-end reads at the Michael Smith Genome Sciences Center on a HiSeq 4000 instrument.

Bisulfite Sequencing Analysis

The adaptor sequences of paired-end 150 bp WGBS raw reads were first trimmed based on the FastQC (v.0.11.8) report using Cutadapt (v.1.9.1) (Martin, 2011). Then the last 15 bp of read1 and first 15 bp

of read2 were cut according to Swift kits manual using Cutadapt (v.1.9.1) (Martin, 2011). Trimmed paired-end reads were then aligned to human reference genome (GRCh38) using BSMAP (v.2.7.4) (Xi and Li, 2009) allowing two mismatches. Methylation levels over each cytosine were then calculated using BSMAP (v.2.7.4) methratio.py scripts. Potential unconverted reads were removed with a customized function incorporated in the methratio.py script (Cokus et al., 2008). Methylation levels over different genomic regions were extracted using a customized Python script. Differentially methylated regions (DMRs) were defined using CT1 (Okae et al., 2018) and WIBR3 (Theunissen et al., 2016) with customized R script over promoters and CpG islands regions. Promoters were defined as upstream 1 kb and downstream 200 bp of transcription start site. Chromosome Y was excluded from the DMR analysis. Regions with coverage (C + T count) greater than 50 in both samples were kept. p values were calculated with Fisher's exact test and then adjusted with the Benjamini-Hochberg procedure (FDR).

Additional thresholds were then applied. To identify regions with CT1-specific CpG island methylation, we required (1) FDR < 0.05, (2) a $\geq$25% absolute difference in CpG methylation level between CT1 hTSCs and WIBR3 hESCs, and (3) $\geq$50 CTs mapped over the region in all eight bisulfite sequencing samples. To identify regions with hESC-specific promoter methylation, we required (1) FDR < 0.05, (2) a $\geq$50% absolute difference in CpG methylation level between CT1 hTSCs and WIBR3 hESCs, and (3) $\geq$50 CTs mapped over the region in all 8 samples.

ELF5 Methylation Analysis

The ELF5 promoter was amplified using primers described previously (Lee et al., 2016). Further details are contained in the Supplemental Experimental Procedures.

Alterations to Images

Brightness and contrast of light microscopy images was uniformly altered in some figures to enhance clarity.

Ethical Permissions

All experiments were approved by the McGill University Faculty of Medicine institutional review board and the CIHR Stem Cell Oversight Committee.

Data and Code Availability

RNA-seq and bisulfite sequencing data have been deposited to the Gene Expression Omnibus database under the accession number GSE152104.

SUPPLEMENTAL INFORMATION

Supplemental Information can be found online at https://doi.org/10.1016/j.stemcr.2020.06.003.

AUTHOR CONTRIBUTIONS

J.K.C., S.Y.K., I.H., J.S., C.S.R., and H.-W.T. conducted the experiments. S.Y.K. and Y.G. conducted the bioinformatic analysis. H.O. and T.A. provided hTSC lines and technical advice. T.F.D., W.L., and W.A.P. supervised the experiments and analysis.

ACKNOWLEDGMENTS

We thank the Goodman Cancer Research Center Flow Cytometry core, the SickKids Center for Applied Genomics Facility, the La Jolla Institute for Allergy and Immunology Sequencing Core, and the Canada Michael Smith Genome Sciences Center at BC Cancer for their dedicated service. We thank the Rudolph Jaenisch (MIT) and Thorold Theunissen (Washington University) labs for providing WIBR3 OCT4-ΔPE-GFP hESCs, and the Amander Clark lab (UCLA) for providing UCLA1 hESCs. This work was funded by the New Frontiers in Research Fund (NFRF) grant NFRFE-2018-00883 and the Canadian Institutes of Health Research (CIHR) project grant PJT-166169 to W.A.P., the Zhejiang Provincial Natural Science Foundation of China, LQ20C060004 to W.L., and PJT-165996 to T.F.D. J.K.C. was supported by a Fonds de recherche Santé Québec graduate fellowship. J.K.C. and I.H. were supported by studentships from the McGill University Faculty of Medicine.

Received: December 8, 2019
Revised: June 2, 2020
Accepted: June 3, 2020
Published: July 2, 2020

REFERENCES

Alison, R.H., Lewis, D.J., and Montgomery, C.A. (1987). Ovarian choriocarcinoma in the mouse. Vet. Pathol. *24*, 226–230.

Amita, M., Adachi, K., Alexenko, A.P., Sinha, S., Schust, D.J., Schulz, L.C., Roberts, R.M., and Ezashi, T. (2013). Complete and unidirectional conversion of human embryonic stem cells to trophoblast by BMP4. Proc. Natl. Acad. Sci. U S A *110*, E1212–E1221.

Barbaux, S., Gascoin-Lachambre, G., Buffat, C., Monnier, P., Mondon, F., Tonanny, M.B., Pinard, A., Auer, J., Bessieres, B., Barlier, A., et al. (2012). A genome-wide approach reveals novel imprinted genes expressed in the human placenta. Epigenetics *7*, 1079–1090.

Cambuli, F., Murray, A., Dean, W., Dudzinska, D., Krueger, F., Andrews, S., Senner, C.E., Cook, S.J., and Hemberger, M. (2014). Epigenetic memory of the first cell fate decision prevents complete ES cell reprogramming into trophoblast. Nat. Commun. *5*, 5538.

Chazaud, C., and Yamanaka, Y. (2016). Lineage specification in the mouse preimplantation embryo. Development *143*, 1063–1074.

Chen, H.C., Hodgen, G.D., Matsuura, S., Lin, L.J., Gross, E., Reichert, L.E., Jr., Birken, S., Canfield, R.E., and Ross, G.T. (1976). Evidence for a gonadotropin from nonpregnant subjects that has physical, immunological, and biological similarities to human chorionic gonadotropin. Proc. Natl. Acad. Sci. U S A *73*, 2885–2889.

Cokus, S.J., Feng, S., Zhang, X., Chen, Z., Merriman, B., Haudenschild, C.D., Pradhan, S., Nelson, S.F., Pellegrini, M., and Jacobsen, S.E. (2008). Shotgun bisulphite sequencing of the *Arabidopsis* genome reveals DNA methylation patterning. Nature *452*, 215–219.

De Paepe, C., Cauffman, G., Verloes, A., Sterckx, J., Devroey, P., Tournaye, H., Liebaers, I., and Van de Velde, H. (2013). Human trophectoderm cells are not yet committed. Hum. Reprod. *28*, 740–749.

Diaz Perez, S.V., Kim, R., Li, Z., Marquez, V.E., Patel, S., Plath, K., and Clark, A.T. (2012). Derivation of new human embryonic stem cell lines reveals rapid epigenetic progression in vitro that can be prevented by chemical modification of chromatin. Hum. Mol. Genet. *21*, 751–764.

Dong, C., Beltcheva, M., Gontarz, P., Zhang, B., Popli, P., Fischer, L.A., Khan, S.A., Park, K.M., Yoon, E.J., Xing, X., et al. (2020). Derivation of trophoblast stem cells from naive human pluripotent stem cells. Elife *9*. https://doi.org/10.7554/eLife.52504.

Gao, X., Nowak-Imialek, M., Chen, X., Chen, D., Herrmann, D., Ruan, D., Chen, A.C.H., Eckersley-Maslin, M.A., Ahmad, S., Lee, Y.L., et al. (2019). Establishment of porcine and human expanded potential stem cells. Nat. Cell Biol. *21*, 687–699.

Guo, G., Stirparo, G.G., Strawbridge, S., Yang, J., Clarke, J., Li, M.A., Myers, S.M., Ozel, B.N., Nichols, J., and Smith, A. (2020). Trophectoderm potency is retained exclusively in human naive cells. bioRxiv https://doi.org/10.1101/2020.02.04.933812.

Guo, G., von Meyenn, F., Rostovskaya, M., Clarke, J., Dietmann, S., Baker, D., Sahakyan, A., Myers, S., Bertone, P., Reik, W., et al. (2017). Epigenetic resetting of human pluripotency. Development *144*, 2748–2763.

Haider, S., Meinhardt, G., Saleh, L., Kunihs, V., Gamperl, M., Kaindl, U., Ellinger, A., Burkard, T.R., Fiala, C., Pollheimer, J., et al. (2018). Self-renewing trophoblast organoids recapitulate the developmental program of the early human placenta. Stem Cell Reports *11*, 537–551.

Hamada, H., Okae, H., Toh, H., Chiba, H., Hiura, H., Shirane, K., Sato, T., Suyama, M., Yaegashi, N., Sasaki, H., et al. (2016). Allele-specific methylome and transcriptome analysis reveals widespread imprinting in the human placenta. Am. J. Hum. Genet. *99*, 1045–1058.

Kunath, T., Yamanaka, Y., Detmar, J., MacPhee, D., Caniggia, I., Rossant, J., and Jurisicova, A. (2014). Developmental differences in the expression of FGF receptors between human and mouse embryos. Placenta *35*, 1079–1088.

Lee, C.Q., Gardner, L., Turco, M., Zhao, N., Murray, M.J., Coleman, N., Rossant, J., Hemberger, M., and Moffett, A. (2016). What is trophoblast? A combination of criteria define human first-trimester trophoblast. Stem Cell Reports *6*, 257–272.

Lee, C.Q.E., Turco, M.Y., Gardner, L., Simons, B.D., Hemberger, M., and Moffett, A. (2018). Integrin alpha2 marks a niche of trophoblast progenitor cells in first trimester human placenta. Development *145*. https://doi.org/10.1242/dev.162305.

Li, Z., Kurosawa, O., and Iwata, H. (2019). Establishment of human trophoblast stem cells from human induced pluripotent stem cell-derived cystic cells under micromesh culture. Stem Cell Res Ther *10*, 245.

Maltepe, E., and Fisher, S.J. (2015). Placenta: the forgotten organ. Annu. Rev. Cell Dev. Biol. *31*, 523–552.

Martin, M. (2011). Cutadapt removes adapter sequences from high-throughput sequencing reads. Embnet J. *17*, 3.

Messmer, T., von Meyenn, F., Savino, A., Santos, F., Mohammed, H., Lun, A.T.L., Marioni, J.C., and Reik, W. (2019). Transcriptional heterogeneity in naive and primed human pluripotent stem cells at single-cell resolution. Cell Rep. *26*, 815–824.e4.

Mischler, A., Karakis, V., Mahinthakumar, J., Carberry, C., San Miguel, A., Rager, J., Fry, R., and Rao, B.M. (2019). Two distinct trophectoderm lineage stem cells from human pluripotent stem cells (Preprint). bioRxiv https://doi.org/10.1101/762542.

Nagamatsu, T., Fujii, T., Ishikawa, T., Kanai, T., Hyodo, H., Yamashita, T., Osuga, Y., Momoeda, M., Kozuma, S., and Taketani, Y. (2004). A primary cell culture system for human cytotrophoblasts of proximal cytotrophoblast cell columns enabling in vitro acquisition of the extra-villous phenotype. Placenta *25*, 153–165.

Nakamura, T., Okamoto, I., Sasaki, K., Yabuta, Y., Iwatani, C., Tsuchiya, H., Seita, Y., Nakamura, S., Yamamoto, T., and Saitou, M. (2016). A developmental coordinate of pluripotency among mice, monkeys and humans. Nature *537*, 57–62.

Nguyen, N.M., and Slim, R. (2014). Genetics and epigenetics of recurrent hydatidiform moles: basic science and genetic counselling. Curr. Obstet. Gynecol. Rep. *3*, 55–64.

Niakan, K.K., Han, J., Pedersen, R.A., Simon, C., and Pera, R.A. (2012). Human pre-implantation embryo development. Development *139*, 829–841.

Nichols, J., and Smith, A. (2009). Naive and primed pluripotent states. Cell Stem Cell *4*, 487–492.

Niwa, H., Toyooka, Y., Shimosato, D., Strumpf, D., Takahashi, K., Yagi, R., and Rossant, J. (2005). Interaction between Oct3/4 and Cdx2 determines trophectoderm differentiation. Cell *123*, 917–929.

Okae, H., Chiba, H., Hiura, H., Hamada, H., Sato, A., Utsunomiya, T., Kikuchi, H., Yoshida, H., Tanaka, A., Suyama, M., et al. (2014). Genome-wide analysis of DNA methylation dynamics during early human development. PLoS Genet. *10*, e1004868.

Okae, H., Toh, H., Sato, T., Hiura, H., Takahashi, S., Shirane, K., Kabayama, Y., Suyama, M., Sasaki, H., and Arima, T. (2018). Derivation of human trophoblast stem cells. Cell Stem Cell *22*, 50–63 e56.

Pastor, W.A., Chen, D., Liu, W., Kim, R., Sahakyan, A., Lukianchikov, A., Plath, K., Jacobsen, S.E., and Clark, A.T. (2016). Naive human pluripotent cells feature a methylation landscape devoid of blastocyst or germline memory. Cell Stem Cell *18*, 323–329.

Pastor, W.A., Liu, W., Chen, D., Ho, J., Kim, R., Hunt, T.J., Lukianchikov, A., Liu, X., Polo, J.M., Jacobsen, S.E., et al. (2018). TFAP2C regulates transcription in human naive pluripotency by opening enhancers. Nat. Cell Biol. *20*, 553–564.

Pfeffer, P.L. (2018). Building principles for constructing a mammalian blastocyst embryo. Biology (Basel) *7*, 41.

Qin, H., Hejna, M., Liu, Y., Percharde, M., Wossidlo, M., Blouin, L., Durruthy-Durruthy, J., Wong, P., Qi, Z., Yu, J., et al. (2016). YAP induces human naive pluripotency. Cell Rep. *14*, 2301–2312.

Rijlaarsdam, M.A., Tax, D.M., Gillis, A.J., Dorssers, L.C., Koestler, D.C., de Ridder, J., and Looijenga, L.H. (2015). Genome wide DNA methylation profiles provide clues to the origin and pathogenesis of germ cell tumors. PLoS One *10*, e0122146.

Roberts, R.M., Loh, K.M., Amita, M., Bernardo, A.S., Adachi, K., Alexenko, A.P., Schust, D.J., Schulz, L.C., Telugu, B.P., Ezashi, T., et al. (2014). Differentiation of trophoblast cells from human embryonic stem cells: to be or not to be? Reproduction *147*, D1–D12.

Rugg-Gunn, P.J., Ferguson-Smith, A.C., and Pedersen, R.A. (2007). Status of genomic imprinting in human embryonic stem cells as

revealed by a large cohort of independently derived and maintained lines. Hum. Mol. Genet. *16* (Spec No. 2), R243–R251.

Smith, Z.D., Shi, J., Gu, H., Donaghey, J., Clement, K., Cacchiarelli, D., Gnirke, A., Michor, F., and Meissner, A. (2017). Epigenetic restriction of extraembryonic lineages mirrors the somatic transition to cancer. Nature *549*, 543–547.

Soejima, H., and Higashimoto, K. (2013). Epigenetic and genetic alterations of the imprinting disorder Beckwith-Wiedemann syndrome and related disorders. J. Hum. Genet. *58*, 402–409.

Stirparo, G.G., Boroviak, T., Guo, G., Nichols, J., Smith, A., and Bertone, P. (2018). Integrated analysis of single-cell embryo data yields a unified transcriptome signature for the human pre-implantation epiblast. Development *145*, dev158501.

Takahashi, S., Okae, H., Kobayashi, N., Kitamura, A., Kumada, K., Yaegashi, N., and Arima, T. (2019). Loss of p57(KIP2) expression confers resistance to contact inhibition in human androgenetic trophoblast stem cells. Proc. Natl. Acad. Sci. U S A *116*, 26606–26613.

Takashima, Y., Guo, G., Loos, R., Nichols, J., Ficz, G., Krueger, F., Oxley, D., Santos, F., Clarke, J., Mansfield, W., et al. (2014). Resetting transcription factor control circuitry toward ground-state pluripotency in human. Cell *158*, 1254–1269.

Tanaka, S., Kunath, T., Hadjantonakis, A.K., Nagy, A., and Rossant, J. (1998). Promotion of trophoblast stem cell proliferation by FGF4. Science *282*, 2072–2075.

Theunissen, T.W., Friedli, M., He, Y., Planet, E., O'Neil, R.C., Markoulaki, S., Pontis, J., Wang, H., Iouranova, A., Imbeault, M., et al. (2016). Molecular criteria for defining the naive human pluripotent state. Cell Stem Cell *19*, 502–515.

Theunissen, T.W., Powell, B.E., Wang, H., Mitalipova, M., Faddah, D.A., Reddy, J., Fan, Z.P., Maetzel, D., Ganz, K., Shi, L., et al. (2014). Systematic identification of culture conditions for induction and maintenance of naive human pluripotency. Cell Stem Cell *15*, 471–487.

Thomson, J.A., Itskovitz-Eldor, J., Shapiro, S.S., Waknitz, M.A., Swiergiel, J.J., Marshall, V.S., and Jones, J.M. (1998). Embryonic stem cell lines derived from human blastocysts. Science *282*, 1145–1147.

Turco, M.Y., Gardner, L., Kay, R.G., Hamilton, R.S., Prater, M., Hollinshead, M.S., McWhinnie, A., Esposito, L., Fernando, R., Skelton, H., et al. (2018). Trophoblast organoids as a model for maternal-fetal interactions during human placentation. Nature *564*, 263–267.

Uhlen, M., Fagerberg, L., Hallstrom, B.M., Lindskog, C., Oksvold, P., Mardinoglu, A., Sivertsson, A., Kampf, C., Sjostedt, E., Asplund, A., et al. (2015). Proteomics. Tissue-based map of the human proteome. Science *347*, 1260419.

Uy, G.D., Downs, K.M., and Gardner, R.L. (2002). Inhibition of trophoblast stem cell potential in chorionic ectoderm coincides with occlusion of the ectoplacental cavity in the mouse. Development *129*, 3913–3924.

Xi, Y., and Li, W. (2009). BSMAP: whole genome bisulfite sequence MAPping program. BMC Bioinformatics *10*, 232.

Stem Cell Reports, Volume *15*

Supplemental Information

Naive Human Embryonic Stem Cells Can Give Rise to Cells with a Trophoblast-like Transcriptome and Methylome

Jessica K. Cinkornpumin, Sin Young Kwon, Yixin Guo, Ishtiaque Hossain, Jacinthe Sirois, Colleen S. Russett, Hsin-Wei Tseng, Hiroaki Okae, Takahiro Arima, Thomas F. Duchaine, Wanlu Liu, and William A. Pastor

Figure S1

A.

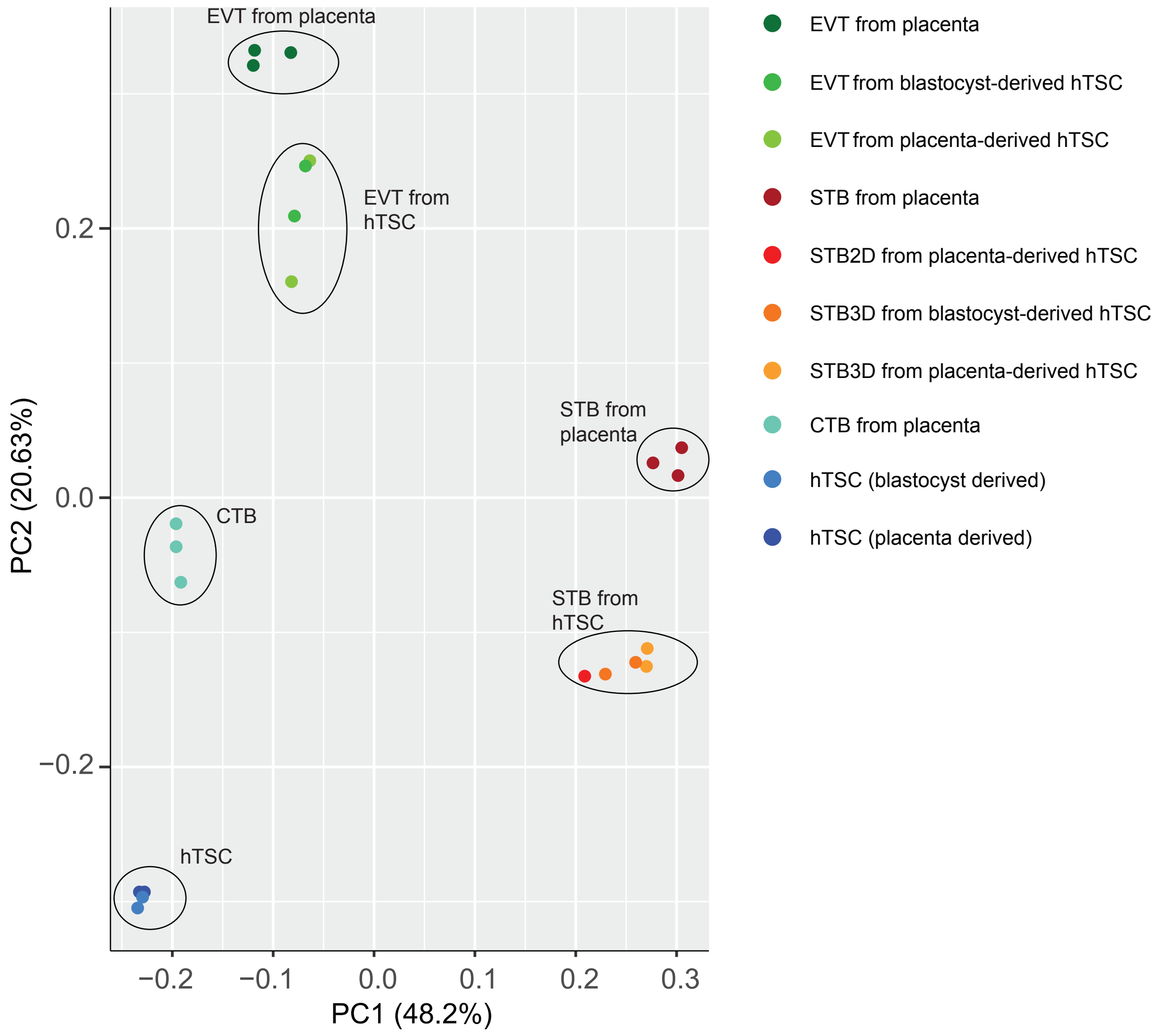
EVT from placenta
EVT from hTSC
STB from placenta
CTB
STB from hTSC
hTSC
0.2
0.0
−0.2
−0.2
−0.1
0.0
0.1
0.2
0.3
PC2 (20.63%)
PC1 (48.2%)
EVT from placenta
EVT from blastocyst-derived hTSC
EVT from placenta-derived hTSC
STB from placenta
STB2D from placenta-derived hTSC
STB3D from blastocyst-derived hTSC
STB3D from placenta-derived hTSC
CTB from placenta
hTSC (blastocyst derived)
hTSC (placenta derived)

B.

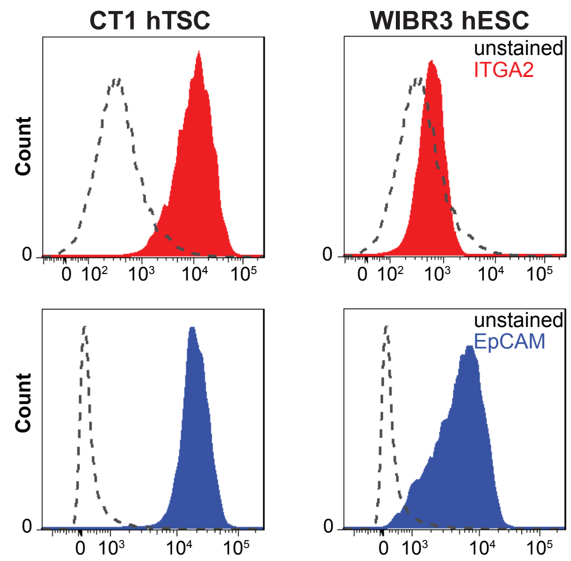
CT1 hTSC
WIBR3 hESC
unstained
ITGA2
Count
0
0 10²
10³
10⁴
10⁵
unstained
EpCAM
Count
0
0 10³
10⁴
10⁵

Figure S1. Principle component analysis of gene expression of different placental cell types. Related to Figure 1. **A.** Principle component analysis was performed using RNA-seq data from (Okae et al., 2018). Cells analyzed include cell types isolated directly from placenta (CTB = cytotrophoblast, STB= syncytiotrophoblast, EVT=extravillous trophoblast) and lines cultured and differentiated *in vitro*. These include blastocyst-derived hTSC lines (BT1 and BT2), placental-derived hTSC lines (CT1 and CT2), as well as EVT and STB derived from these lines. "2D" and "3D" STB refer to two differentiation protocols. **B.** Flow cytometry for ITGA2 and EpCAM in hTSCs and hESCs. Staining profile is indicated relative to unstained control.

Figure S2

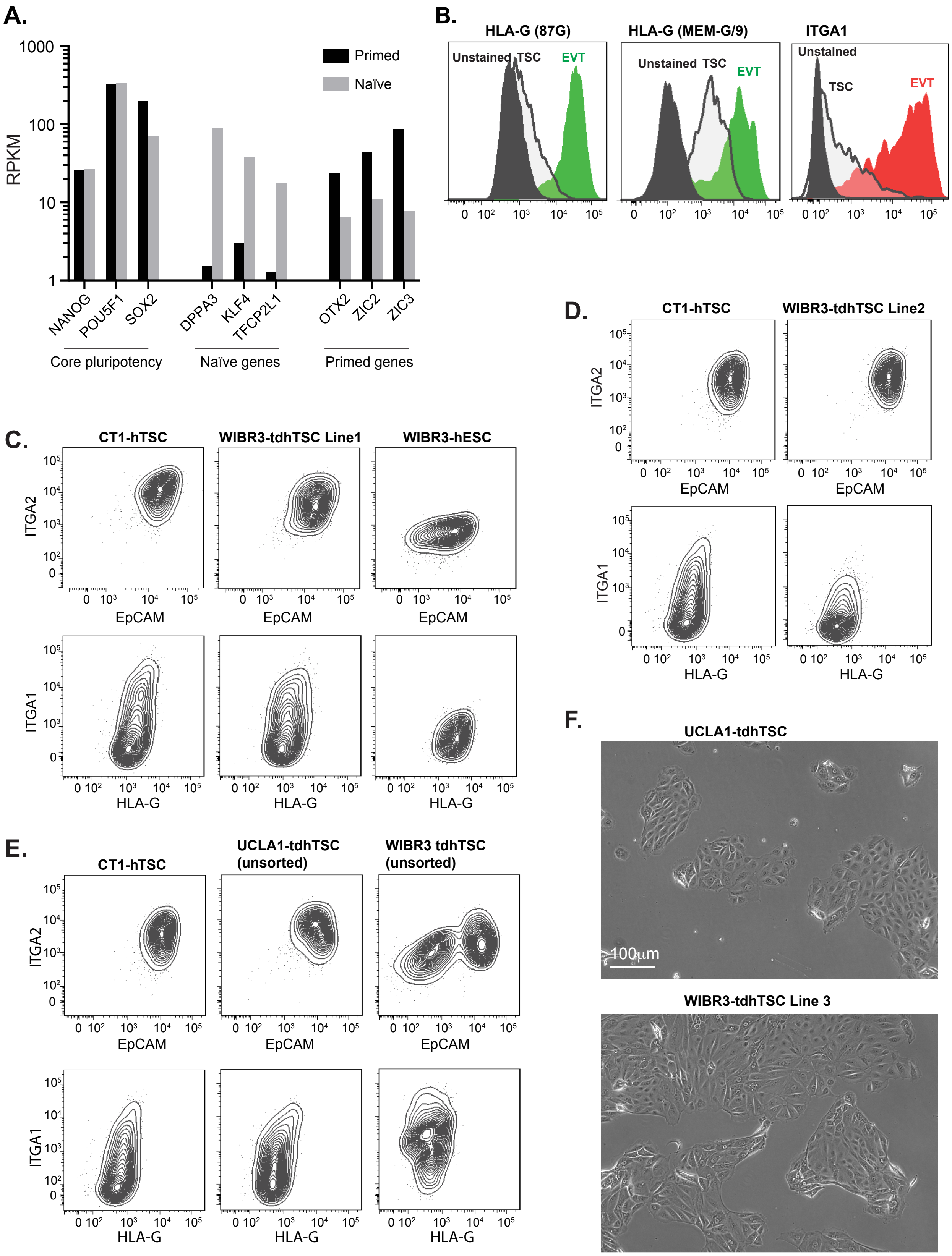
A.
Primed
Naïve
RPKM
1000
100
10
1
NANOG
POU5F1
SOX2
DPPA3
KLF4
TFCP2L1
OTX2
ZIC2
ZIC3
Core pluripotency
Naïve genes
Primed genes
B.
HLA-G (87G)
HLA-G (MEM-G/9)
ITGA1
Unstained
TSC
EVT
C.
CT1-hTSC
WIBR3-tdhTSC Line1
WIBR3-hESC
ITGA2
EpCAM
ITGA1
HLA-G
D.
CT1-hTSC
WIBR3-tdhTSC Line2
E.
CT1-hTSC
UCLA1-tdhTSC (unsorted)
WIBR3 tdhTSC (unsorted)
F.
UCLA1-tdhTSC
100μm
WIBR3-tdhTSC Line 3

Figure S2. Transdifferentiation of hESCs to putative hTSCs and purification via FACS sorting. Related to Figure 2. **A.** Expression of primed and naïve hESC markers in primed and Day 10 naïve cells (which were used as the starting point for generation of WIBR3 tdhTSC Line 1) as measured by RNA-sequencing of one replicate. **B.** Flow cytometry validation of HLA-G and ITGA1 antibodies used in other figures. Note elevated HLA-G and ITGA1 signal in EVT. Representative of n= 5 – 15 independent experiments per antibody **C.** Flow cytometry of WIBR3 tdhTSC Line 1, 16 days after sorting, with CT1 and WIBR3 hESCs as comparisons. Representative of n= 2 (hESCs), and n=3 (WIBR3 tdhTSC L1) independent flow cytometry experiments conducted with these lines. **D.** Flow cytometry profile of WIBR3 tdhTSC Line 2. Representative of n= 3 independent experiments. **E.** UCLA1 and WIBR3 hESCs were reverted and transferred to hTSC media, with flow cytometry conducted 7 days later. Note formation of $ITGA2^{hi}$ $EpCAM^{hi}$ $ITGA1^{lo}$ cells in both lines. Same CT1 control was used in D. and E. **F.** Photographs of sorted $ITGA2^{hi}$ $EpCAM^{hi}$ $ITGA1^{lo}$ UCLA1 tdhTSC Line 1 and WIBR3 tdhTSC Line 3.

Figure S3

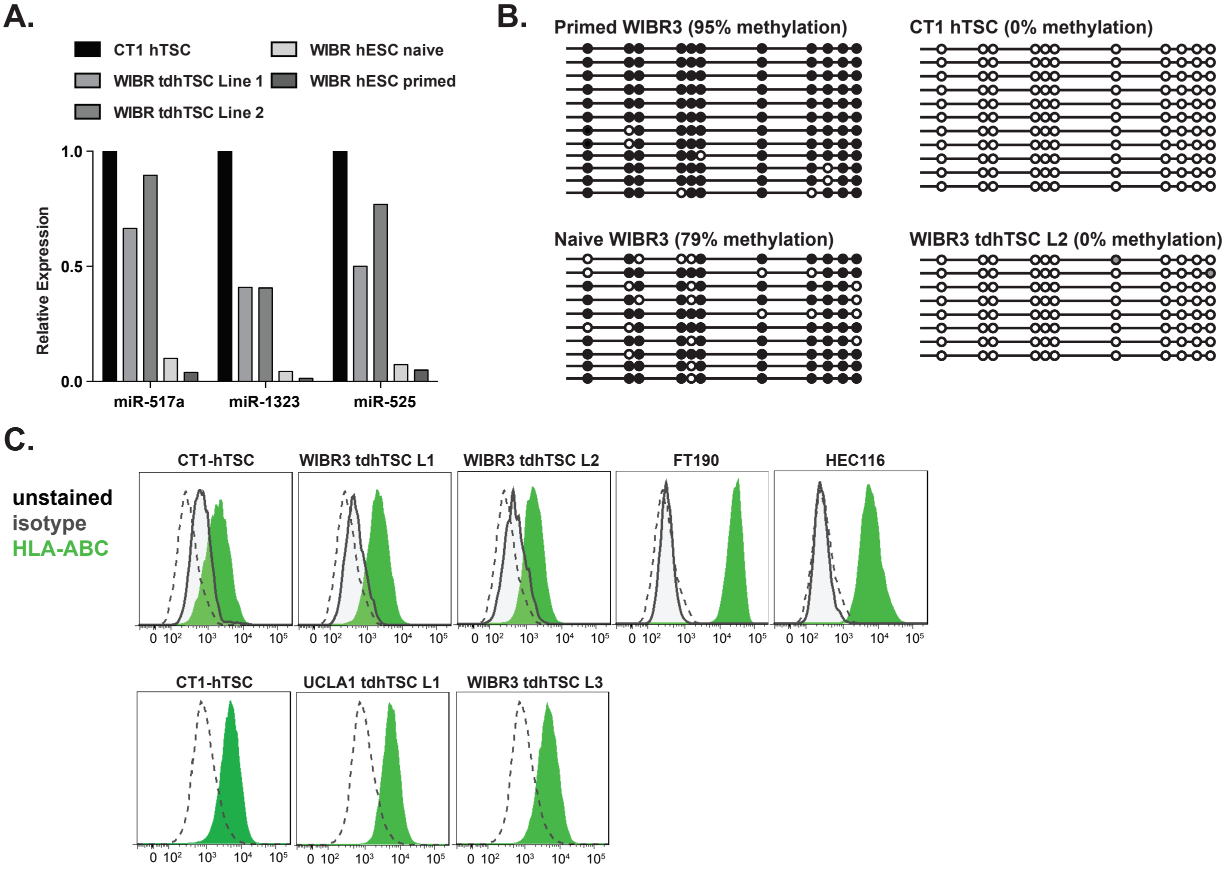

Figure S3. Transdifferentiated hTSCs show hallmarks of placental identity. Related to Figure 3. **A.** RT-PCR of three miRNA that are generated from the placental C19MC transcript. n=1 biological replicate. **B.** Bisulfite PCR of the ELF5 locus for the Primed, Naïve and WIBR3 tdhTSC L2, as well as control CT1 line. Each CG site is indicated with a circle, with an empty circle indicating an unmethylated CG, a black circle indicating methylation, and a gray circle indicating non-informative sequencing. Note modest demethylation in naïve culture and dramatic demethylation upon transdifferentiation. **C.** Flow cytometry for a pan-HLA antibody in CT1, WIBR3 tdhTSC Lines 1 and 2, FT190 and Hec116. Unstained, isotype control, and pan-HLA staining profiles are indicated. Representative of n=2 independent experiments.

Figure S4

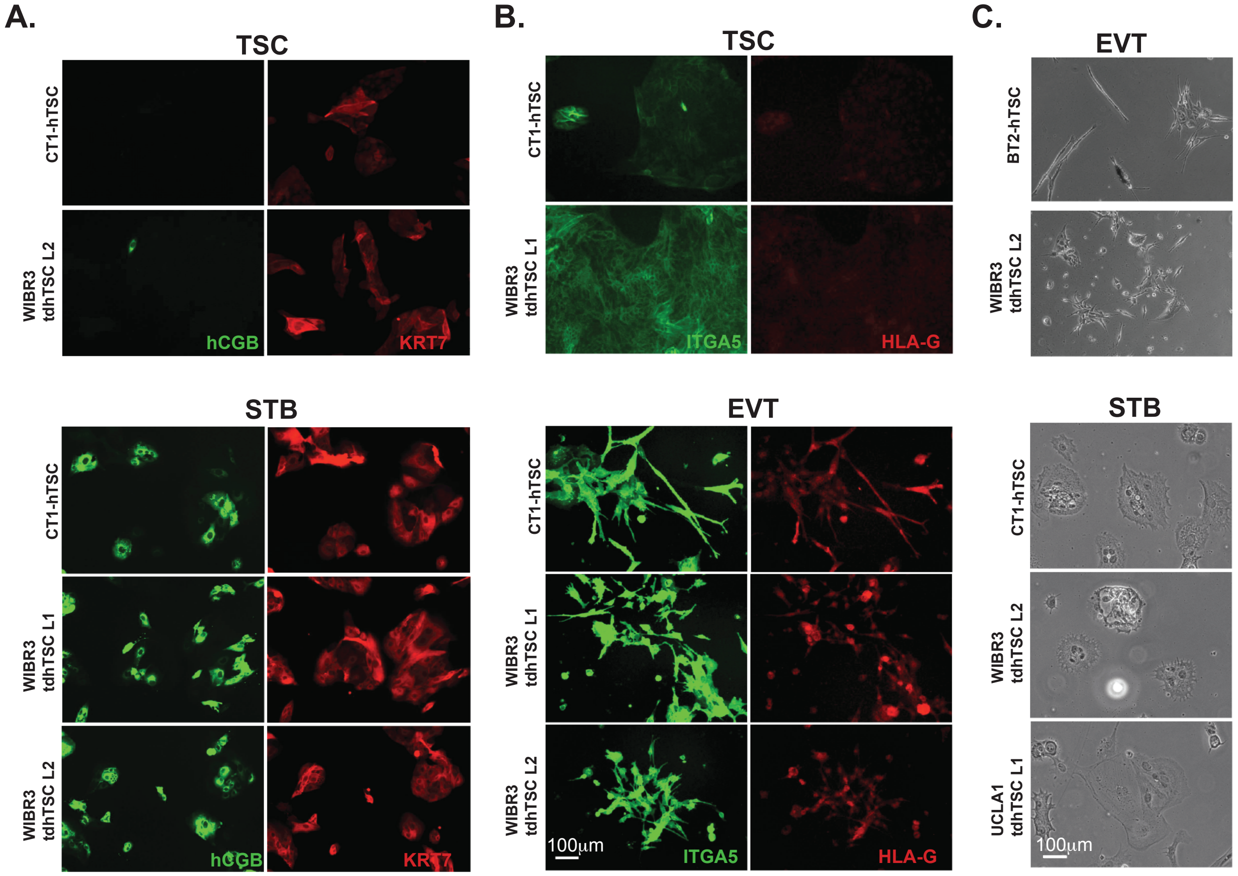

Figure S4. Differentiation capacity of tdhTSCs. Related to Figure 4. **A,B.** Immunofluorescent staining of cells indicated with STB marker (hCGB), EVT markers (ITGA5, HLA-G) or a pan-placental marker (KRT7). Note gain of hCGB upon STB differentiation **(A)** and gain of spindly mesenchymal morphology and increased staining for ITGA5 and HLA-G upon EVT differentiation **(B). C.** Light microscopy photos of EVTs and STBs indicated.

Figure S5

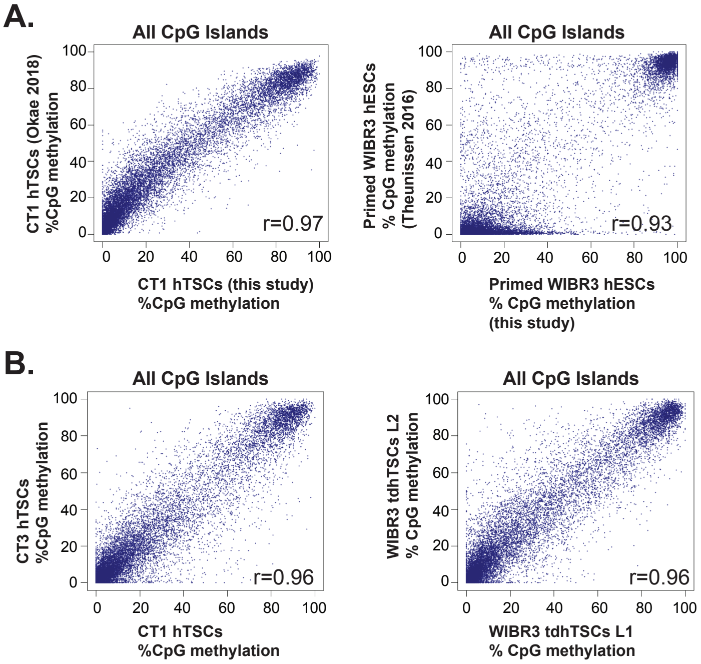

Figure S5. Global methylation patterns of tdhTSC. Related to Figure 5. **A.** Scatterplot showing CpG island methylation for each CpG island in our sample (X-axis) and published data (Y-axis). Note high correlation between new and published data. **B.** Scatterplot showing CpG island methylation for similar samples (CT1 vs. CT3, WIBR3 tdhTSC L1 vs. L2).

Figure S6

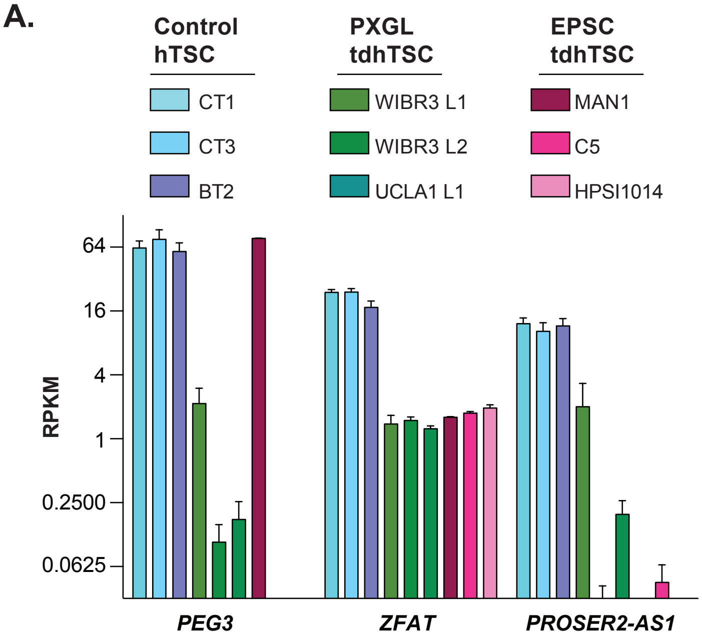

Figure S6. Imprinting abnormalities in tdhTSC. Related to Figure 6. **A.** Expression of the three imprinted genes indicated in control and tdhTSCs. Data for EPS tdhTSCs are from published sources(Gao et al., 2019). n=2 (all tdhTSC lines), n=4 (CT3, BT2) or n=7 (CT1) biological replicates.

Figure S7

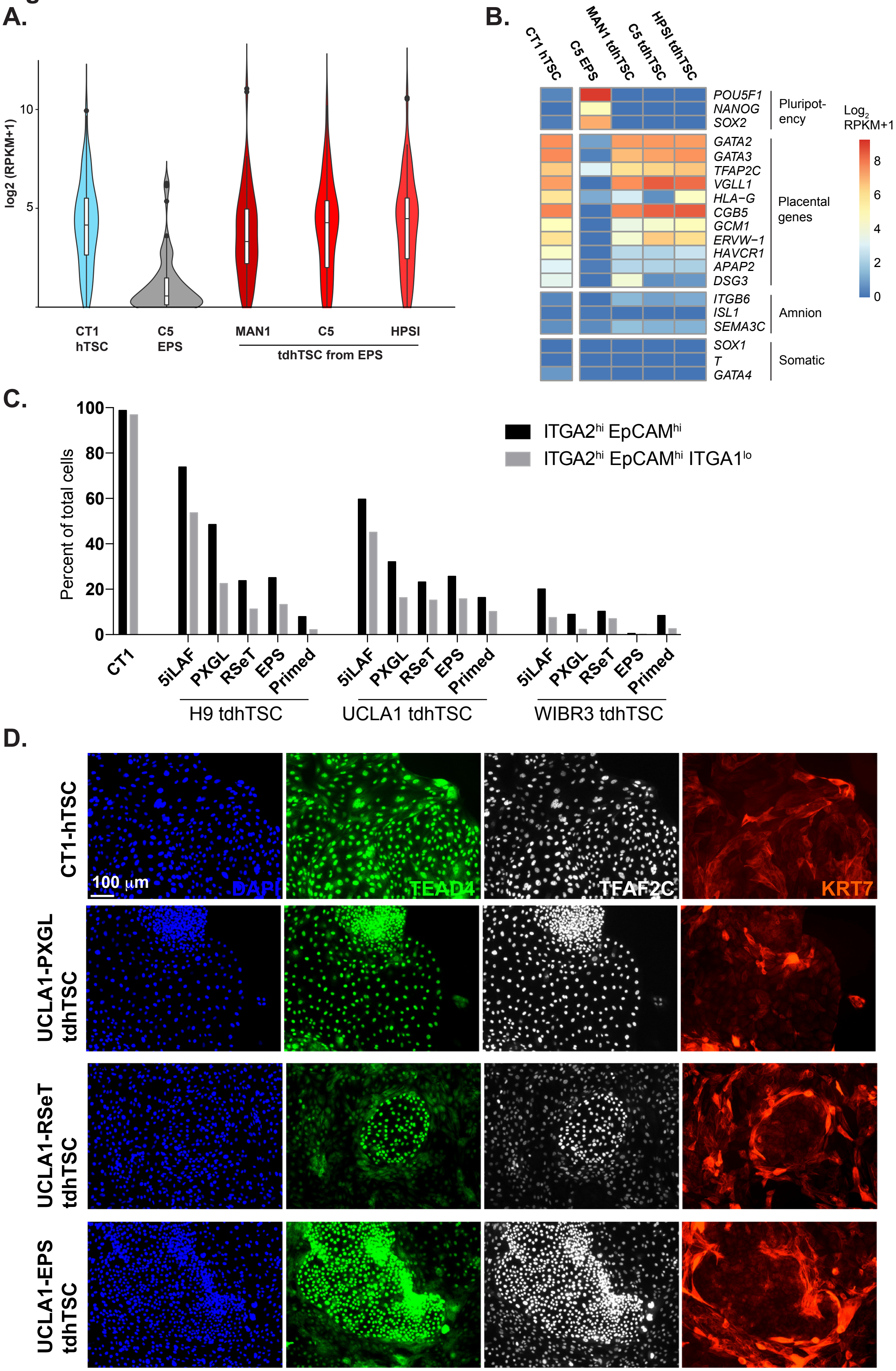
A.
log2 (RPKM+1)
10
5
CT1
hTSC
C5
EPS
MAN1
C5
HPSI
tdhTSC from EPS
B.
CT1 hTSC
C5 EPS
MAN1 tdhTSC
C5 tdhTSC
HPSI tdhTSC
POU5F1
NANOG
SOX2
Pluripot-
ency
GATA2
GATA3
TFAP2C
VGLL1
HLA−G
CGB5
GCM1
ERVW−1
HAVCR1
APAP2
DSG3
Placental
genes
ITGB6
ISL1
SEMA3C
Amnion
SOX1
T
GATA4
Somatic
Log2
RPKM+1
8
6
4
2
0
C.
Percent of total cells
100
80
60
40
20
0
ITGA2hi EpCAMhi
ITGA2hi EpCAMhi ITGA1lo
CT1
5iLAF
PXGL
RSeT
EPS
Primed
H9 tdhTSC
UCLA1 tdhTSC
WIBR3 tdhTSC
D.
CT1-hTSC
UCLA1-PXGL
tdhTSC
UCLA1-RSeT
tdhTSC
UCLA1-EPS
tdhTSC
100 μm
DAPI
TEAD4
TFAF2C
KRT7

Figure S7. Comparative transdifferentiation capacity from different media conditions. Related to Figure 7. **A.** Expression of 89 placental genes (same as in Figure 3D) in tdhTSCs derived from EPS cells. Expression of each gene, using an average of all replicates for a given cell type, is indicated as a single point on the violin plot. n=2 (EPS and tdhTSC) or n=7 (CT1) biological replicates. Data for EPS cells and EPS tdhTSCs are from published sources (Gao et al., 2019). **B.** Expression of markers indicated from RNA-seq data. Data for EPS cells and EPS tdhTSCs are from published sources (Gao et al., 2019). Data from n=2 (EPS and tdhTSC) or n=7 (CT1) biological replicates are averaged. **C.** Percentage of ITGA2^{hi} EpCAM^{hi} and ITGA2^{hi} EpCAM^{hi} ITGA1^{lo} cells after hESCs of the line and starting conditions indicated are cultured in hTSC media for 15 days. n=1 experiment. **D.** Immunofluorescent images of control CT1 hTSCs and tdhTSCs derived from hESCs cultured in naïve (PXGL) and primed-like (EPS, RSeT) conditions. Note uniform staining for the three indicated markers in CT1 and PXGL-derived hTSCs, but a mixture of TEAD4^{hi} and TEAD4^{lo} cells in EPS or RSeT derived tdhTSCs. n=1 experiment.

Supplemental Table Captions

Table S1. STR analysis of WIBR3 hESCs and TSC. Related to Figure 2.

Table S2. Descriptions of transdifferentiations. Related to Figures 2 and 7. Description of circumstances of generation of each cell line, efficiency of production of ITGA2^{hi} EpCAM^{hi} and ITGA2^{hi} EpCAM^{hi} ITGA1^{lo} cells, and figures in which each cell line was used.

Table S3. Sample description and mapping statistics. Related to Figures 3, 5, 6.

Table S4. RPKM of all samples. Related to Figures 3, 5, 6.

Table S5. Expression of trophoblast-specific genes in RNA-seq samples. Related to Figure 3. Expression data used to generate Figure 3D.

Table S6. Methylation data. Related to Figures 5,6. CpG islands with CT1-hTSC-specific methylation, promoters with hESC-specific methylation, and putative gatekeeper genes and their expression levels in different samples are all shown.

Table S7. Genes differentially expressed between control hTSC and tdhTSC. Related to Figure 6. First two panels: Genes that show differential expression between hTSCs (CT1, CT3, BT2) and primed hESCs (WIBR3, UCLA1) are listed, along with fold-change and $\log_2$ read count per million. Positive fold-change value indicates higher expression in hESCs. Third and fourth panels: Genes that show differential expression between control hTSCs (CT1, CT3, BT2) and tdhTSCs (WIBR-tdhTSC Line 1, WIBR3-tdhTSC Line 2, UCLA1 tdhTSC) are listed. Positive fold-change value indicates higher expression in tdhTSCs.

Supplemental Experimental Procedures

Naïve Reversion: Primed hESCs were routinely passaged in TeSR-E8 media (Stemcell Technologies). In the first naïve reversion we conducted (which led to the generation of WIBR3 tdhTSC L1 and L2), primed hESCs were cultured in special primed media (DMEM-F12 supplemented with 15% FBS, 5% KSR, 1X Glutamax, 1X non-essential amino acids, 0.1mM β-mercaptoethanol and 8 ng/ml FGF) on mitomycin C inactivated mouse embryonic fibroblast (MEF) feeder layers for one passage prior naïve reversion. In subsequent reversions, cells were cultured in TeSR-E8 until naïve induction.

In accordance with published protocol (Guo et al., 2017) 200,000 primed hESCs were dissociated into single cells using 30% TrypLE Express and plated onto a MEF feeder layer in special primed media (in the first reversion) or TeSR E8 (in subsequent reversions) supplemented with 10 μM ROCKi at 5% O_2. After 24h, primed hESCs were cultured for 3 days in naïve induction medium (24.5 ml DMEM F-12, 24.5 ml Neurobasal media, 0.25 ml N2, 0.5 ml B27, 1x Glutamax, 150 μM L-ascorbic acid, 0.1mM β-mercaptoethanol, 1μM PD0325901, 10μM ROCKi, 0.75mM vaporic acid and 10ng/ml recombinant hLIF). Following induction, reverting naïve hESCs were cultured in PXGL maintenance medium (24.5 ml DMEM F-12, 24.5 ml Neurobasal media, 0.25 ml N2, 0.5 ml B27, 1xGlutamax, 150 μM L-ascorbic acid, 0.1mM β-mercaptoethanol, 1μM PD0325901, 2μM Gö6983, 10μM ROCKi, 2μM XAV939 and 10ng/ml recombinant hLIF) indefinitely. Reverting naïve cells started showing dome-like morphology with refractive edges 7 days post-induction. We observed that culture with ascorbate (described as optional in previous protocols(Guo et al., 2016)) enhanced subsequent transdifferentiation (data not shown).

Culture of RSet cells was conducted with a commercially available reagent (Stemcell Technologies 05975) and manufacturer protocol. EPS cells were generated and cultured by published protocol(Gao et al., 2019). Cells were cultured in RSeT or EPS media for 11 days before culture in hTSC media.

Flow cytometry: Cells were dissociated with TrypLE Express (Gibco 12604) and quenched with Soybean trypsin inhibitor (Gibco 17075), then passed over a 70µm filter to remove aggregates. Cells were then centrifuged 3 minutes at 200xg and resuspended in 1ml FACS buffer (1xPBS, 1%BSA) and counted.

Cells were stained with appropriate fluorescent antibodies at a concentration of 1µg antibody per one million cells in a volume of 1ml. When fewer cells were used, antibody and volume were scaled down accordingly. Cells were incubated with antibody for 20 minutes in the dark at 4°C, then centrifuged, washed with 1ml of FACS buffer, resuspended in 300µl FACS buffer and analyzed. DAPI was included immediately before flow to distinguish dead cells. Flow cytometry was performed using a BD FACSAria Fusion instrument, FACS with a BD LSRFortessa instrument, and analysis was performed using FlowJo v10.

A table indicating which antibodies were used and in which figures is shown below:

EPCAM conj. BV711	Biolegend	324239	Figure 1C, 2E, 2H, 7A, 7B, S1B, S2C-E
ITGA1 (Integrin alpha 1) conj. APC	Biolegend	328313	Figure 2E, 2H, 4C, S2B-E
ITGA2 (Integrin alpha 2) conj. 594	R&D	FAB1233T	Figure 1C, 2E, 2H, 7A, 7B, S1B, S2C-E
ITGA6 (Integrin alpha 6) conj. APC/Fire 750	Biolegend	313631	Figure 7B
ITGB6 (Integrin beta 6) conj. APC	Miltenyi	130-111-454	Figure 7B,C
HLA-G (Major histocompatibility complex-G) conj. 488	Biolegend	335917	Figure 2E, 2H, S2B-E
HLA-G (Major histocompatibility complex-G) conj. PE	Abcam	24384	Figure 4C, S2B
HLA-ABC (Major histocompatibility complex-ABC) conj. 488	Biolegend	311415	Figure S3C
IgG anti-mouse	Cell signalling	61656	Figure S3C

Immunofluorescence*:* Cells were plated and grown on glass coverslips coated with extracellular matrix medium appropriate for the cell type or differentiation. Cells were fixed in 4% PFA, 0.75xPBS for 15 minutes at room temperature, then washed twice with 1xPBS. They were then permeabilized and blocked by treatment with blocking buffer (0.1% Triton X, 5% Donkey serum, 1xPBS) for 30 minutes, then washed twice with 1xPBS 0.05% Tween-20. Cells were incubated with indicated concentration of primary antibody in blocking buffer for 1-2 hours at room temperature. Cells were then washed twice with 1xPBS 0.05% Tween-20. Secondary antibody incubation was performed by incubation for one hour with Invitrogen ALEXA-Fluor antibodies. Cells were washed once more with 1xPBS 0.05% Tween-20 containing DAPI (Sigma), then mounted with Invitrogen Prolong GOLD to preserve the imaging. Images were taken with Zeiss-Axiovert or Invitrogen-EVOS and fluorescence was adjusted uniformly across experiments.

Antibodies and respective concentrations are indicated below:

ITGA5 (Integrin alpha 5)	dilution: 1:1000	Abcam	150361
hCGB (human Chorionic Gonadotropin)	dilution: 1:1000	Abcam	131170
KRT7 (Cytokeratin 7) conj. 594	dilution: 1:500	Biolegend	601603
HLA-G (Major histocompatibility complex) conj.PE	dilution: 1:500	Abcam	24384
TFAP2C (Transcription factor AP-2)	dilution: 1:1000	Abcam	76007
TEAD4 (Transcription enhancer factor)	dilution: 1:500	Abcam	58310

Real time PCR analysis of gene expression: RNA isolation was performed using the RNAzol total RNA protocol (Sigma). cDNA synthesis was performed using Froggabio SensiFAST cDNA synthesis kit using 500ng total RNA/10µl reaction following manufacturer settings. Each reaction mix is then diluted to 50 µl with Rnase DNase free H_2O. 1µL of each cDNA reaction mix, corresponding to 10ng of starting RNA, was used in each 15 µl reaction of Invitrogen PowerUp SYBR green mix containing 1µM of primer mix. Quantification and analysis were performed on the QuantStudio5 instrument.

Primers:

ITGA2:	F:5'-ATGAAAACCAACATGAGCCTCG-3'	R:5'-GATTCCCACATTGCTGTGCC-3'
GCM1:	F:5'-TGAACACAGCACCTTCCTCC-3'	R:5'-CGCCTTCCTGGAAAGACCAA-3'
HLA-G:	F:5'-GCCAAGGATGGTGGTCATGG-3'	R:5'-GCGGCGCTGAAATACCTCAT-3'
CGB7:	F:5'-CGCACCAAGGATGGAGATGT-3'	R:5'-GAAGCATCTCCCTGGATGCC-3'
TFAP2C	F-5'- TGCACGATCAGACAGTCATTC	R-5'-GTAGAGCTGAGGAGCGACAATC-3'
GATA3	F-5'- TGCAGGAGCAGTATCATGAAGCCT -3'	R-5'-GCATCAAACAACTGTGGCCAGTGA-3'
XAGE3	F-5'-CCGAGGAGAAGTGTACCACC-3'	R-5'-TGCAGGATCCCGACTTTCAG-3'
HAVRC1	F-5'-GGCGTATATTGTTGCCGTGT-3'	R-5'-GACGGTTGGAACAGTTGTGA-3'
ELF5	F-5'- GACGCTGAAGAAAGCAAGGC-3'	R-5'- CCCATTCCAGAATGCCACAG-3'
ITGB6	F-5'-GAAGAAATTGCCAACCCTTG-3'	R-5'TGTCAATGGCAAAATGTGCT-3'

The TFAP2C, GATA3 and ELF5 primers were taken from published sources(Lee et al., 2016).

ELF5 Methylation analysis: 500 ng of genomic DNA was bisulfite-converted using EZ DNA Methylation-Lightning Kit (Zymo) as per instructions. Converted DNA was eluted in 10 µL of M-elution buffer.

The ELF5 promoter was amplified using primers described previously(Lee et al., 2016). We used a nested PCR strategy in which the locus was first amplified with primer set #1 (forward:5'-GGAAATGATGGATATTGAATTTGA-3', reverse:5'-CAATAAAAATAAAAACACCTATAACC-3'). Each 20 µL PCR reaction was carried out using 10 µl 2X Zymotaq premix, 0.5 µl forward primer, 0.5 µl reverse primer, 8 µL H20, and 1 µL converted DNA, with PCR cycling conditions: 95°C 10:00, 35x (95°C 30s, 45°C 30s, 72°C 30s), 72°C 7:00). 1µl of the reaction product was then amplified using identical conditions and cycles with a second set of primers (forward:5'-GAGGTTTTAATATTGGGTTTATAATG-3', reverse:5'-ATAAATAACACCTACAAACAAATCC-3').

PCR products were purified with a MinElute PCR purification kit (Qiagen), using manufacturer protocols with the addition of a second elution step in which the eluant is passed over the column a second time. PCR product was cloned into pGEM-T Easy Vector (Promega) using a 1:3 plasmid:insert molar ratio, transformed into DH5α competent bacteria, cultured and miniprepped using the Presto Mini Plasmid Kit (FroggaBio).

C19 microRNA cluster expression analysis: Total RNA was extracted using RNAzol RT (Bioshop) followed by isopropanol precipitation. DNase treatment was then performed on all the total RNA samples with TURBO DNase (Thermo Fisher) following manufacturer's instructions. To quantify mature miRNAs, the miScript kit (Qiagen) was used for reverse transcription using 500ng of total RNA input and diluted 1 in 4 for subsequent RT-PCR reactions. QuantiTect SYBR Green PCR kit (Qiagen) was used for miRNA quantification, with customized forward primers for miRNAs, primer assay for the control U6 snRNA (Qiagen MS00033740), and the kit supplied universal primer as the reverse primer. The cycling condition for RT-PCR is: (95°C 15 min, 45x (94°C 15 s, 55°C 30 s, 70°C 30 s), 95°C 15 s). The following primers were used:

miR-1323: TCAAAACTGAGGGGCATTTTCT

miR-525-5p: CTCCAGAGGGATGCACTTTCT
miR-517a-3p: ATCGTGCATCCCTTTAGAGTGT

Supplemental References

Gao, X., Nowak-Imialek, M., Chen, X., Chen, D., Herrmann, D., Ruan, D., Chen, A.C.H., Eckersley-Maslin, M.A., Ahmad, S., Lee, Y.L.*, et al.* (2019). Establishment of porcine and human expanded potential stem cells. Nat Cell Biol *21*, 687-699.

Guo, G., von Meyenn, F., Rostovskaya, M., Clarke, J., Dietmann, S., Baker, D., Sahakyan, A., Myers, S., Bertone, P., Reik, W.*, et al.* (2017). Epigenetic resetting of human pluripotency. Development *144*, 2748-2763.

Guo, G., von Meyenn, F., Santos, F., Chen, Y., Reik, W., Bertone, P., Smith, A., and Nichols, J. (2016). Naive Pluripotent Stem Cells Derived Directly from Isolated Cells of the Human Inner Cell Mass. Stem Cell Reports *6*, 437-446.

Lee, C.Q., Gardner, L., Turco, M., Zhao, N., Murray, M.J., Coleman, N., Rossant, J., Hemberger, M., and Moffett, A. (2016). What Is Trophoblast? A Combination of Criteria Define Human First-Trimester Trophoblast. Stem Cell Reports *6*, 257-272.

Okae, H., Toh, H., Sato, T., Hiura, H., Takahashi, S., Shirane, K., Kabayama, Y., Suyama, M., Sasaki, H., and Arima, T. (2018). Derivation of Human Trophoblast Stem Cells. Cell Stem Cell *22*, 50-63 e56.
